# Leveraging synthetic and genetic data to improve epidemic forecasting

**DOI:** 10.1371/journal.pcbi.1014630

**Published:** 2026-08-27

**Authors:** Dave Osthus, Alexander C. Murph, Emma E. Goldberg, Lauren J. Beesley, William M. Fischer, Nidhi Parikh, Lauren A. Castro

**Affiliations:** 1 Statistics Group, Los Alamos National Laboratory, Los Alamos, New Mexico, United States of America; 2 Theoretical Biology and Biophysics Group, Los Alamos National Laboratory, Los Alamos, New Mexico, United States of America; 3 Computational Intelligence and Modeling Group, Los Alamos National Laboratory, Los Alamos, New Mexico, United States of America; University of Washington, UNITED STATES OF AMERICA

## Abstract

Forecasting infectious disease outbreaks is hard. Forecasting emerging infectious diseases with limited historical data is even harder. In this paper, we investigate ways to improve emerging infectious disease forecasting when little pathogen-specific training data are available. Specifically, we explore two sources of information that may be available near the start of an emerging disease outbreak: synthetic data and genetic information. For this investigation, we conducted an experiment where we trained deep learning models on different combinations of real and synthetic data, both with and without genetic information, to explore how these models compare when forecasting COVID-19 cases for US states. All models are developed with an eye towards forecasting the next pandemic. We find that models trained with synthetic data have better forecast accuracy than models trained on real data alone, and models that use genetic variants have better forecast accuracy compared to those that do not. All models outperformed a baseline persistence model, a benchmark that proved challenging for many real-time COVID-19 case forecasting models, and multiple models outperformed the COVIDHub-4_week_ensemble. This paper demonstrates the value of these underutilized sources of information and provides a blueprint for forecasting future pandemics.

## Author summary

Forecasting emerging infectious diseases is difficult because, by definition, little or no historical data are available for the pathogen of interest. This lack of training data is a major barrier to using flexible machine learning models in emerging disease forecasting settings. In this paper, we investigate two sources of information likely to be available near the start of a future outbreak: synthetic outbreak data and viral genetic information. Using COVID-19 as a test case, we train transformer-based models on historical respiratory disease data, synthetic outbreaks, and SARS-CoV-2 variant information to forecast weekly cases in U.S. states. Models trained with synthetic data outperform those trained on historical data alone, and models trained on both real and synthetic data perform best overall. We also find that using variant-attributable cases improves forecasts relative to forecasting total cases directly. We show these models were competitive with the best real-time COVID-19 case forecasting models. Together, these results show that synthetic data and genomic surveillance are practical, underused tools for epidemic forecasting. They offer a path toward scalable forecasting systems that can be deployed early in the next pandemic, when reliable forecasts are most needed and hardest to produce.

## 1 Introduction

Over the past decades, infectious disease forecasting has transformed from an academic curiosity into a critical tool for public health preparedness and response. This growth has been driven by advances in modeling [2–4] and data availability [5], and by the recognition that timely forecasts can guide resource allocation, inform policy, reduce the cost burden associated with emerging health threats, and improve public health outcomes [6]. Yet forecasting remains inherently difficult: challenges include noisy and incomplete data [7], lags in reporting [8], reflexive human behavior [9], uneven policy decisions [10], and pathogen evolution [11,12]. These obstacles are particularly pronounced during rapidly evolving outbreaks, where even the best models struggle to keep pace [1]. The COVID-19 pandemic brought these struggles into acute focus [13]. On one hand, it marked an unprecedented mobilization of forecasting talent and infrastructure [14,15]; on the other, it revealed gaps—particularly in integrating real-time signals, quantifying uncertainty, and anticipating the emergence of new viral dynamics.

Among the success stories of the COVID-19 response was the rapid and widespread adoption of genomic surveillance [16]. SARS-CoV-2 genomes were sequenced and shared globally at unprecedented scale and speed, offering a near real-time continuously updated feed of the virus’s evolution [17,18]. With relatively low technical barriers and declining sequencing costs, genomic surveillance is poised to remain a cornerstone in future outbreaks. Crucially, it enabled the early identification of new variants, which often preceded observable surges in cases [19]. This makes variant tracking a potential leading indicator—a rare and powerful feature in infectious disease forecasting. Looking ahead, this stream of high-resolution, biologically meaningful data offers a promising direction for improving forecast model accuracy.

In general, forecasting systems perform best when three conditions are met: (1) the system’s underlying mechanisms are well-characterized, (2) there is sufficient historical data to train models, and (3) future trends don’t deviate too significantly from past ones [20]. Emerging infectious diseases typically violate the first two conditions, and sometimes all three. While mechanistic models—such as compartmental models [21], agent-based models [22], or phylodynamic models [23]—can capture transmission and/or evolutionary dynamics, they are often difficult to fit to incomplete and noisy real-time data, and have often been outperformed by their more flexible statistical or machine learning model counterparts in real-time forecasting exercises [24]. Flexible statistical and machine learning models are therefore promising tools for operational forecasting, but their flexibility can also be a weakness when the outbreak of interest differs substantially from the data used to develop or train them.

In this paper, we seek to develop forecasting models that are accurate, scalable, and easily deployed in a pandemic setting where little to no historical data are available for the pathogen of interest. We consider transformer-based deep learning models for forecasting because, although often costly to train, they are cheap to deploy, can maintain strong performance on new data without frequent retraining; they allow scaling to many data subsets (e.g., geographies), and offer high performance ceilings.

This modeling framework sits within a broader literature on deep learning for epidemic analysis and forecasting, including multilayer perceptrons, convolutional neural networks, and recurrent neural networks such as long short-term memory networks [25–27]. Relevant work also includes graph neural network approaches for spatio-temporal epidemic forecasting, which model geographic regions as connected nodes with coupled disease dynamics [28,29], as well as deep-learning-based phylodynamic methods that use genetic sequences or phylogenetic trees directly for epidemiological parameter inference, model selection, and characterization of transmission dynamics [30,31]. In contrast to the latter, our use of genetic data is intentionally more aggregated: we use variant labels and variant-attributable case counts as surveillance summaries rather than modeling genome sequences or phylogenetic trees directly.

For the forecasting problem considered here, the central challenge is training data. To learn, these models require large amounts of training data, yet for an emerging pathogen, by definition, pathogen-specific datasets are limited. In this setting, essentially two types of training data are available at or near outbreak onset: historical outbreak data from other pathogens and synthetic data. Neither data source will perfectly represent the pathogen of concern, yet both are available *at outbreak onset* and so can be used to train deep learning models before substantial pathogen-specific surveillance data have accumulated.

Historical outbreak datasets are finite, restricted to outbreaks that have occurred and were measured, and thus represent only a portion of the space of possible outbreaks. Recent work has demonstrated success in incorporating data from different pathogens and surveillance streams to improve forecasting [4,32]; this strategy is enabled by the public dissemination of public health data, e.g., [33]. Thus, while historical data do not represent the emerging pathogen (the forecast target), they do represent ostensibly relevant data, including data reporting vagaries, useful for model training. This mirrors the logic of pretraining and transfer learning in neural networks, where models trained on related source domains can learn reusable representations that improve performance or reduce data requirements in a target domain [34,35].

Synthetic datasets address many of the shortcomings of historical data. They are infinite in number (in principle): their generation is constrained primarily by compute resources. Furthermore, they can represent a diversity of outbreaks limited only by the choice of simulation parameters; measurement noise and biases can be added separately to mimic realistic surveillance systems. Researchers have recently shown success of models trained exclusively on synthetic outbreak data [36–38]. This being said, the realism of these outbreaks and the potential gains of using synthetic data in modeling are restricted by the fidelity of the simulator and by the measurement error processes.

Synthetic data must conform to real or prospective data as it is (or will be) measured. Many infectious disease models based on first principles (e.g., compartmental models or agent-based models) can straightforwardly generate time series of case-counts, hospitalizations and deaths. To make use of viral genetic variant information, as we do here, a synthetic infectious disease simulator must model outbreaks at the variant level, and aggregate variant data to produce “observed case-count” time series. In this paper we make use of one such simulator, *MutAntiGen* [39] (discussed in detail in Section 4). This simulator produces both time series of total observed cases (referred to as total cases, or TCs) as well as time series of each constituent variant (referred to as variant-attributable cases, or VACs).

Given this framing, we seek in this work to answer the following five research questions:

**Q1:** *Does training with real data or synthetic data produce better forecast performance?*
**Q2:** *Does joint training with real and synthetic data improve COVID-19 case forecasts relative to training with either source individually?*
**Q3:** *Two questions comparing synthetic TC and VAC training data:*
  **Q3a:** *Does training with synthetic VAC data improve COVID-19 forecasts relative to training with synthetic TC data?*
  **Q3b:** *Do models with matched training data and input data outperform models with mismatched training data and input data?*
**Q4:** *Does including SARS-CoV-2 variant information improve COVID-19 case forecasts?*
**Q5:** *How do these forecasts compare to real-time COVID-19 case forecasts?*

To answer these five questions, we fit and compare eight forecasting models that differ in both the data used to train the models and the inputs to the models, described in Table 1. While eight models are defined in Table 1, they correspond to four different fitted deep learning models trained on different data sets (real, synthetic TCs, synthetic VACs, or real plus synthetic), each applied to two different model input types (TCs or VACs).

**Table 1:** Forecast model configurations by training data source and input time series. The model naming convention is “M(training data, input type)” where training data can be r = real, st = synthetic total cases (TCs), sv = synthetic variant-attributable cases (VACs), and a = all sources (real plus synthetic total cases plus synthetic variant-attributable cases) and input types can be t = total cases and v = variant-attributable cases.

Model	Training Data	Input Time Series
**M(0)**	N/A (persistence baseline)	N/A
**M(r,t)**	Real	TC
**M(st,t)**	Synthetic, TC	TC
**M(sv,t)**	Synthetic, VAC	TC
**M(a,t)**	Real + Synthetic	TC
**M(r,v)**	Real	VAC
**M(st,v)**	Synthetic, TC	VAC
**M(sv,v)**	Synthetic, VAC	VAC
**M(a,v)**	Real + Synthetic	VAC

For concreteness, models M(r,t) and M(r,v) use the same fitted deep learning model (the model trained only with real training data), but M(r,t) predicts total cases directly and M(r,v) forecasts each variant-attributable case directly and sums up the individual forecasts; additional forecasting details are provided in Section 4.3.2.

The models defined in Table 1 allow for a systematic evaluation of how synthetic data and genetic information can improve forecast accuracy by comparing forecast performance of pairs of models. Specifically,

- (**Q1**) If models trained with synthetic data outperform models trained with real data, we would expect M(st,t) > M(r,t) and M(sv,v) > M(r,v), where M(A) > M(B) means M(A) outperformed M(B).
- (**Q2**) If joint training with real and synthetic data improves COVID-19 case forecasts relative to training with either source individually, we would expect M(a,t)> [M(r,t), M(st,t)], and M(a,v)> [M(r,v), M(sv,v)].
- (**Q3a**) If training with synthetic VACs improves COVID-19 case forecasts relative to training with synthetic TCs, we would expect M(sv,v) > M(st,t).
- (**Q3b**) If models with matched training data and input data outperform models with mismatched training data and input data, we would expect M(st,t) > M(sv,t) and M(sv,v) > M(st,v).
- (**Q4**) If including SARS-CoV-2 variant information improves COVID-19 case forecasts, we would expect M(r,v) > M(r,t), M(st,v) > M(st,t), M(sv,v) > M(sv,t), and M(a,v) > M(a,t).
- Q5 will be evaluated with external models later.

We next present the results of the exercise, including direct answers to all research questions stated above. We then discuss implications, limitations, and future directions of work. Details on the study design, data sources, synthetic data generation, forecasting models, and evaluation metrics are provided in Section 4.

## 2 Results

We evaluated weekly 1–4 week ahead forecasts of COVID-19 cases for U.S. states and Puerto Rico from June 2020 through December 2022. The study used COVID-19 as a retrospective test case for pandemic forecasting models trained without COVID-19 data, allowing us to evaluate whether historical respiratory disease data, synthetic outbreak data, and SARS-CoV-2 variant information could improve forecasts under emerging-outbreak constraints. Full study-design details are provided in Section 4.

We first provide high-level findings from our exercise in Section 2.1. Then in Section 2.2 we directly answer the research questions stated in Section 1. Evaluation metrics are defined in Section 4.

### 2.1 Overview of results

**Fig 1:**
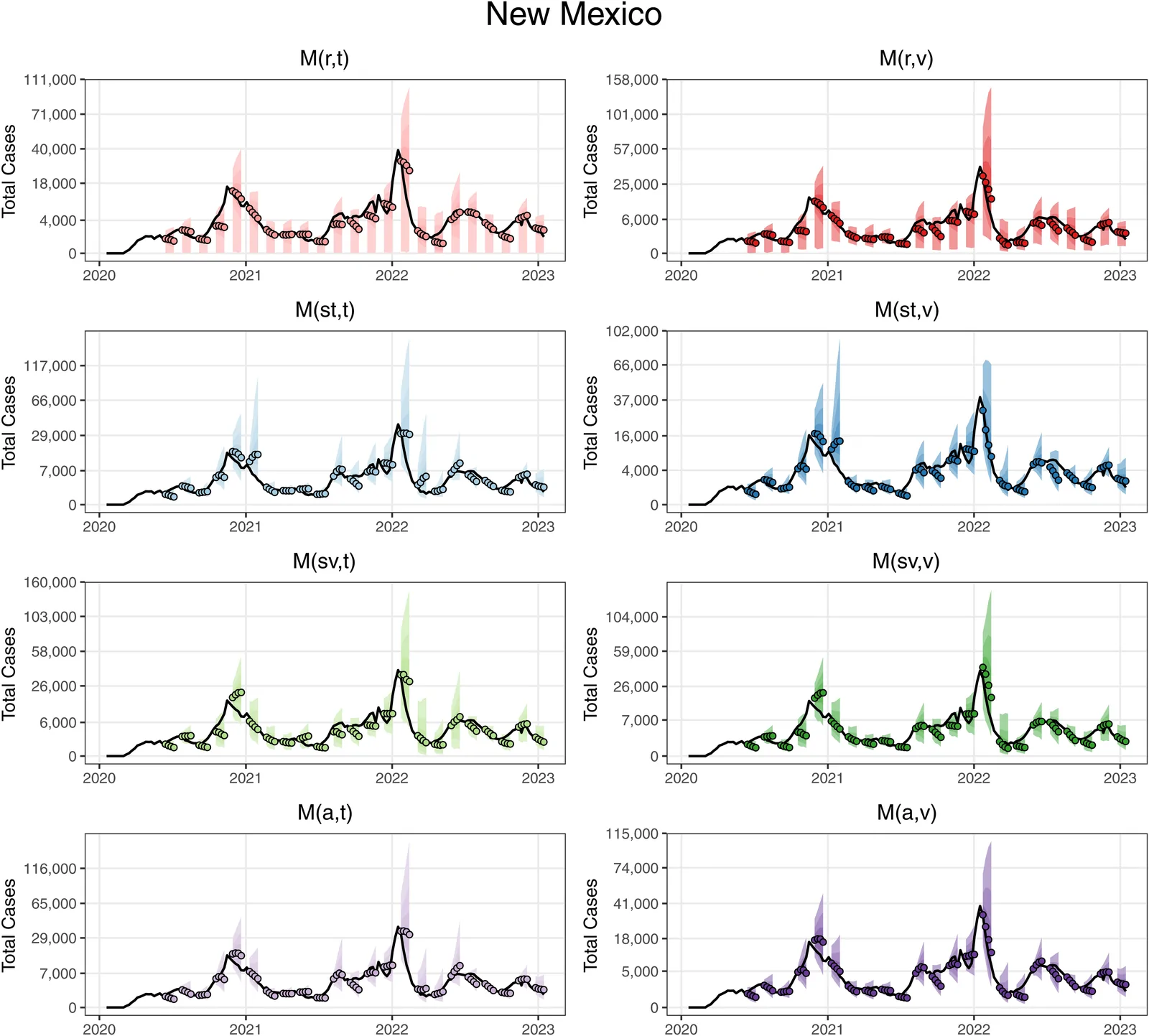
Selected 1-week-ahead through 4-week-ahead forecasts for New Mexico for all models. Black line: total cases time series. Colored points: median forecast cases. Ribbons mark the 50%, 80%, and 95% forecast intervals. Note: y-axis is on a square root scale to better see the low-case-count forecasts.

Fig 1 shows selected forecasts for New Mexico for all models. As expected, forecast interval widths increase with increasing horizon *h*. The forecasts for models trained on only real data (M(r,t) and M(r,v)) have noticeably low quantile estimates at the 0.025 level. The real data used for training are fairly noisy, which may have led to this forecast behavior. The other notable trend is the forecasts made at the peak of the Omicron (BA.1) wave in early 2022. The models that take TCs as input, M(.,t) (left column of Fig 1), produced flat to mildly dropping forecasts at the peak in early 2022, while the models that take VACs as inputs, M(.,v) (right column of Fig 1), correctly produced a steep drop in their forecasts. Fig 1 illustrates that there are systematic differences in the forecast models that break down along training data and model input lines.

**Fig 2:**
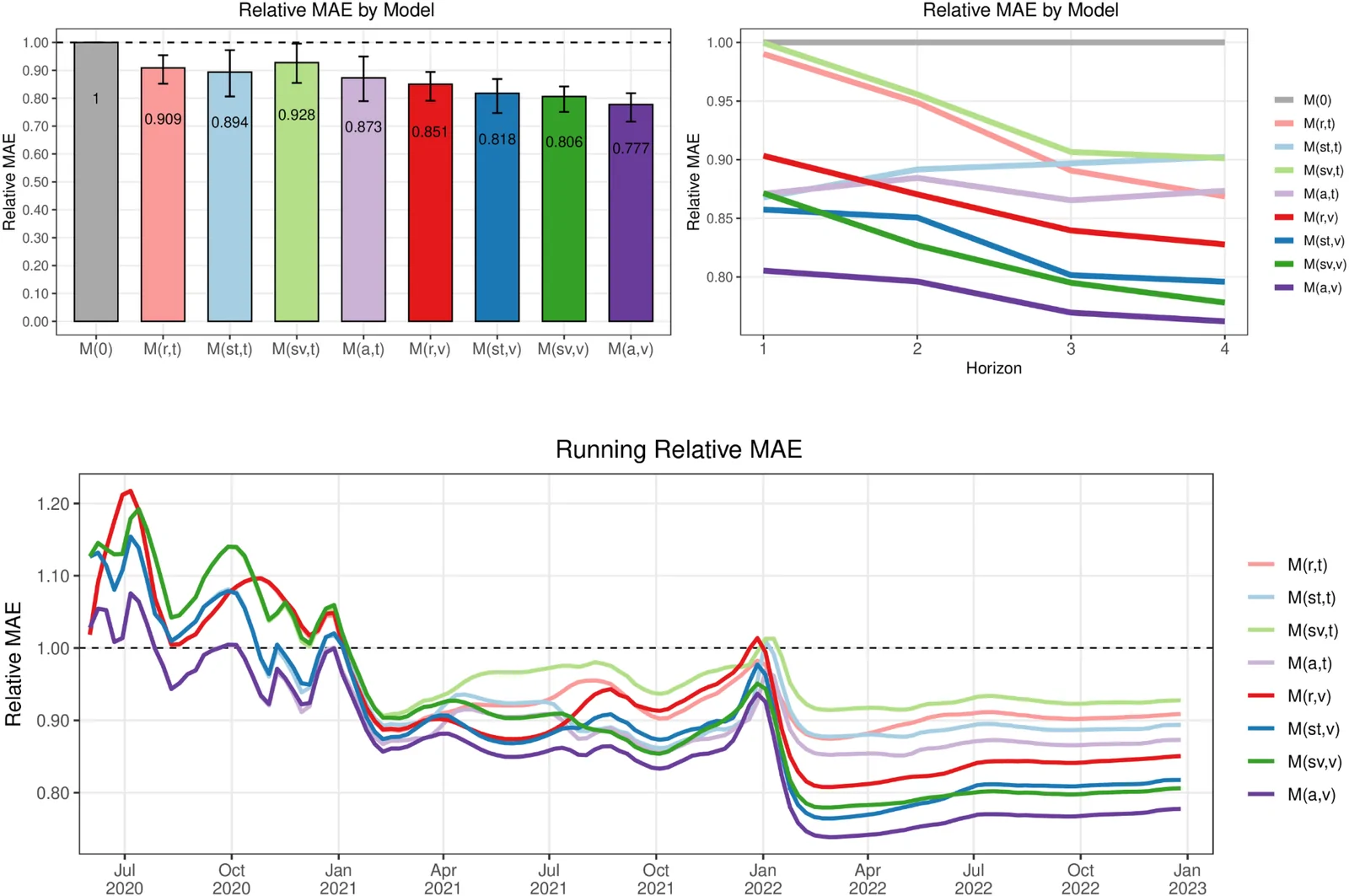
(Top left) Overall relative mean absolute error (rMAE) with 95% confidence intervals as error bars. Error bars were constructed via bootstrapping. (Top right) Relative MAE by forecast horizon. (Bottom) Running relative MAE. The running relative MAE for each date is the relative MAE if the evaluation period ran from June 1st, 2020 through the x-axis date.

Fig 2 shows rMAE results. Overall, we see all models had better MAE than the baseline, the worst being M(sv,t) with 0.928 rMAE and the best being M(a,v) with 0.777 rMAE. Uncertainty intervals are 95% confidence intervals, obtained via bootstrapping (see S1 Text for details). Each model had rMAE less than or equal to 1 for all forecast horizons. Fig 2 Bottom shows a running relative MAE where the rMAE on a given date corresponds to the evaluation of all forecasts available between June 1st, 2020 through the x-axis date. It is clear the results depend on the evaluation period, as models perform differently throughout different phases of the pandemic. That said, for all evaluation end times after January 2021, all models had an rMAE less than 1 (except for a momentary blip for a few models around January 2022).

**Fig 3:**
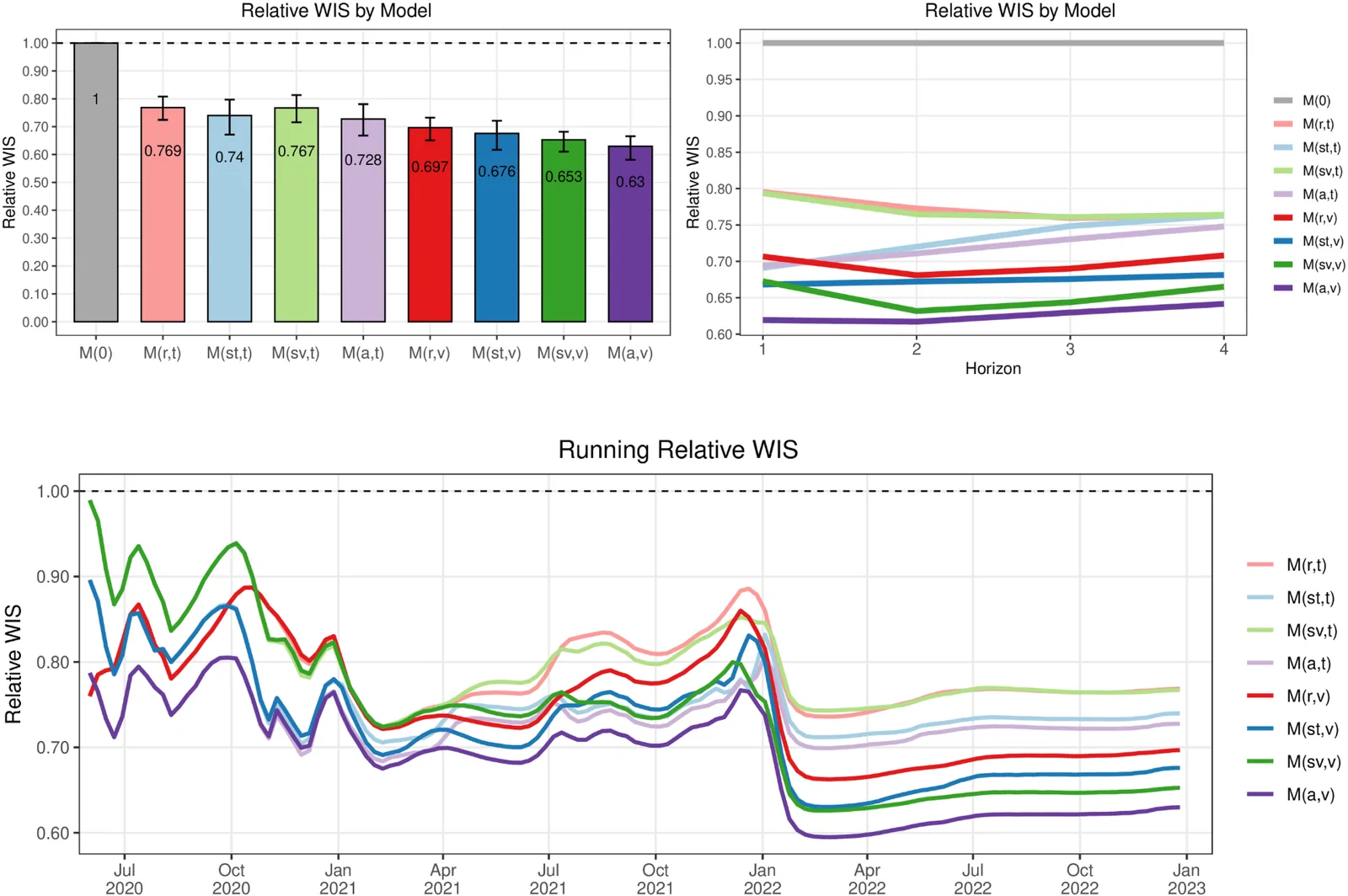
(Top left) Overall relative weighted interval score (WIS) with 95% confidence intervals as error bars. Error bars were constructed via bootstrapping. (Top right) Relative WIS by forecast horizon, and (Bottom) running relative WIS. The running relative WIS for each date is the relative WIS if the evaluation period ran from June 1st, 2020 through the x-axis date.

Fig 3 shows results for WIS relative to a persistence model. Overall, all models had rWIS less than 1 ranging from 0.769 (M(r,t)) to 0.63 (M(a,v)). Each model also had rWIS less than 1 (in fact, less than or equal to 0.80) for all considered forecast horizons. The running relative WIS is less than 1 for all models and all evaluation end dates between June 2020 through December 2022 providing strong evidence that all models demonstrated better forecast performance as measured by WIS compared to M(0).

**Fig 4:**
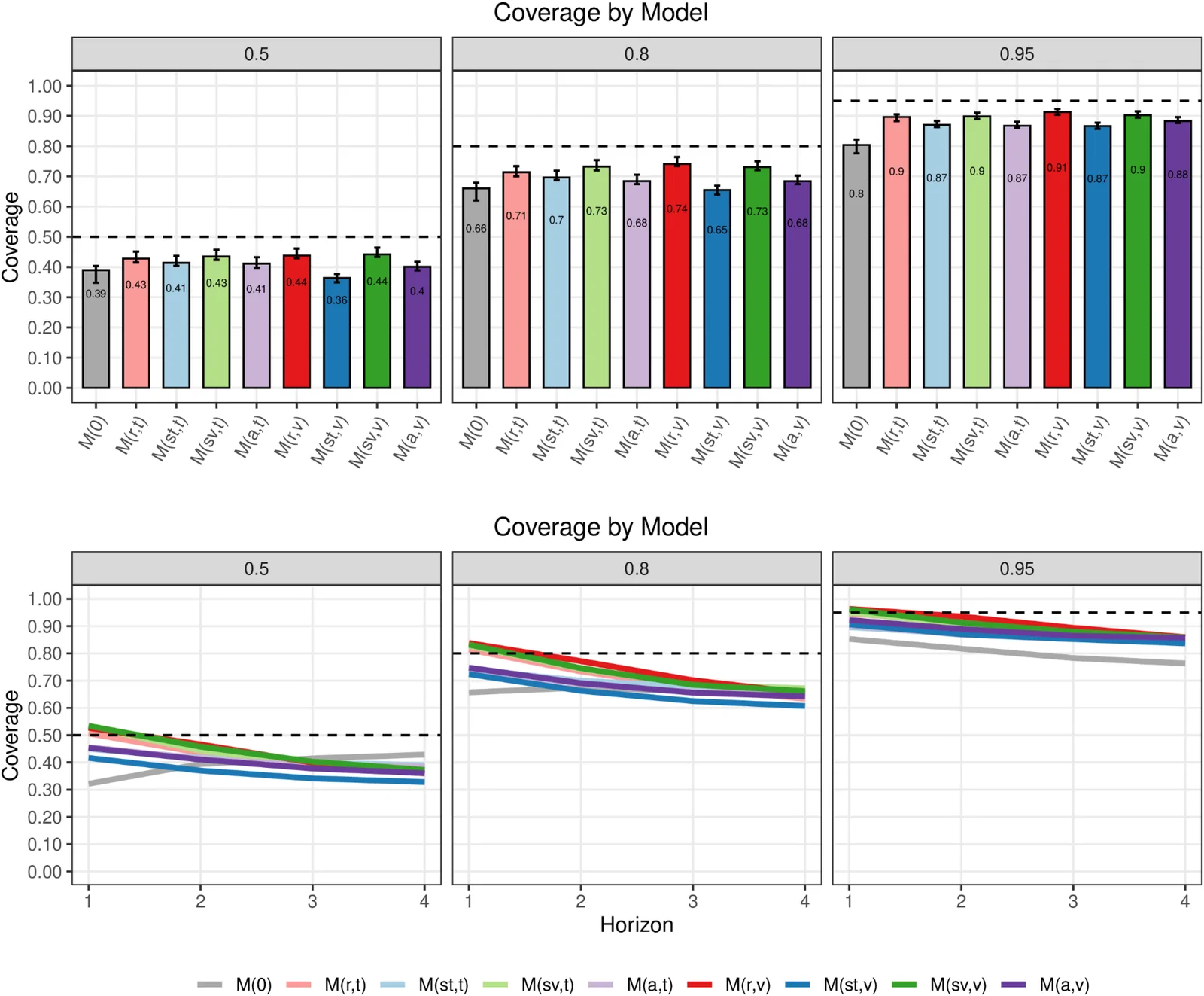
(Top) Overall empirical coverages for 50%, 80%, and 95% forecast intervals. (Bottom) Empirical coverage by horizon. The dashed horizontal line indicates the nominal coverage. Undercoverage exists for all models and horizons.

Fig 4 shows the empirical coverage for all models, overall and by horizon. All models exhibit undercoverage (i.e., observations fell outside their predictive intervals more often than expected), a common occurrence in COVID-19 forecasting [1]. All models showed empirical coverage similar to that of a persistence model for 50% forecast intervals, but empirical coverages closer to nominal than the persistence model for 95% forecast intervals. When we examine empirical coverage by forecast horizon (Fig 4 Bottom), we generally see that all non-persistence models have near nominal coverage at horizon 1, but that empirical coverage drifts below nominal coverage as horizon increases to 4 weeks ahead.

### 2.2 Answering the research questions

We now directly answer our research questions laid out in Section 1.

#### 2.2.1 Q1: Does training with real data or synthetic data produce better forecast performance?.

**Fig 5:**
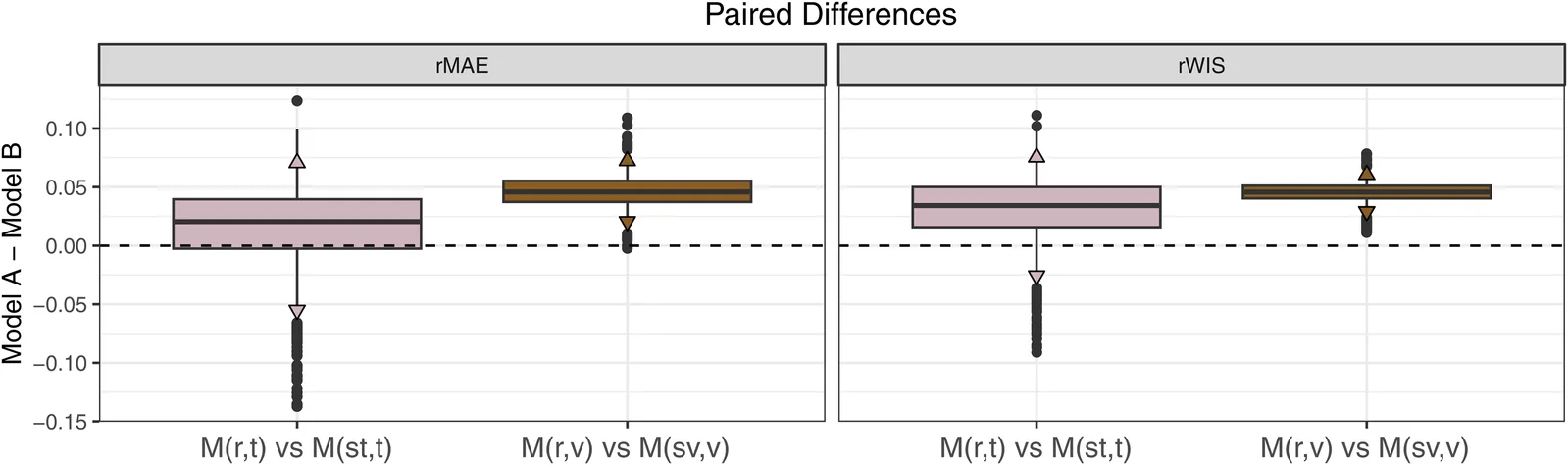
Distribution of 5,000 bootstrapped model differences for rMAE and rWIS. Triangles indicate the 2.5 and 97.5 percentiles. Presented results show Model A - Model B (on the x-axis it is displayed as M(A) vs M(B)). For instance, M(r,t) vs M(st,t) in the rMAE panel presents the results of M(r,t)’s rMAE - M(st,t)’s rMAE, across all bootstrap samples. Positive numbers indicate Model B performed better than Model **A.** Fairly strong evidence is shown that models trained with synthetic data alone performed better than models trained with real data alone.

There is fairly strong evidence that training with synthetic data alone produces better forecast performance than training with real data alone. That evidence is presented in Fig 5, which shows the paired differences of rMAE and rWIS across 5,000 bootstrapped samples. The model comparisons were M(r,t) vs M(st,t) and M(r,v) vs M(sv,v). We see that in over 75% of bootstrapped samples, model M(st,t) outperformed M(r,t) in both rMAE and rWIS (i.e., the lower edge of the box of the box plot is at or above 0), while M(sv,v) outperformed M(r,v) in nearly 100% of bootstrapped samples.

#### 2.2.2 Q2: Does joint training with real and synthetic data improve COVID-19 case forecasts relative to training with either source individually?.

**Fig 6:**
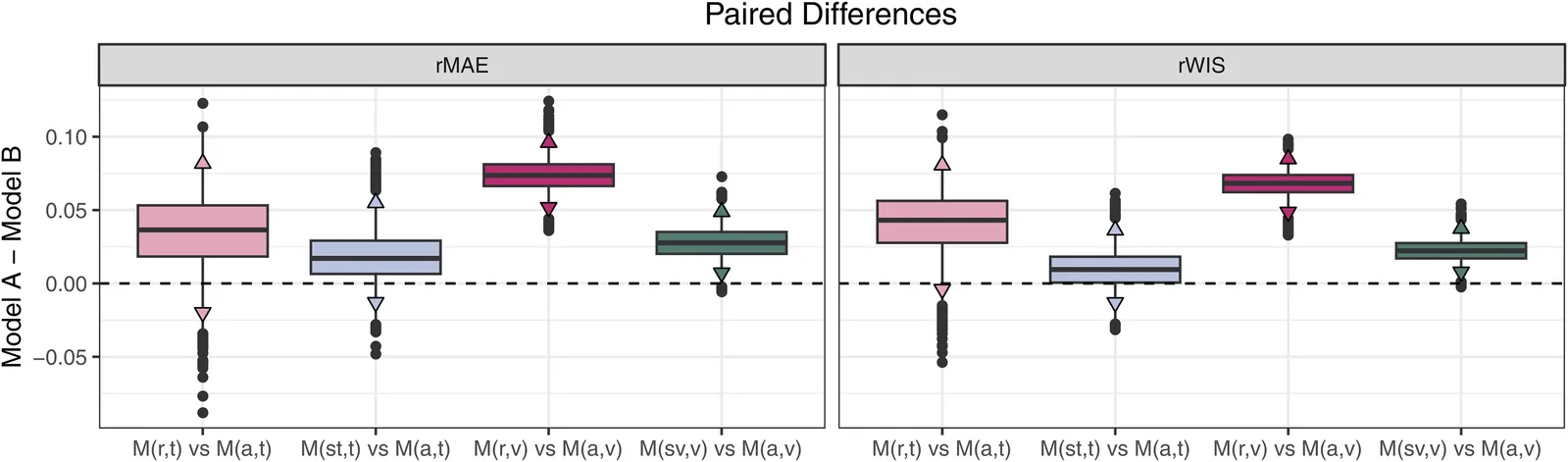
Distribution of 5,000 bootstrapped model differences for rMAE and rWIS. Triangles indicate the 2.5 and 97.5 percentiles. Presented results show Model A - Model B (on the x-axis it is displayed as M(A) vs M(B)). For instance, M(r,t) vs M(a,t) in the rMAE panel presents the results of M(r,t)’s rMAE - M(a,t)’s rMAE, across all bootstrap samples. Positive numbers indicate Model B performed better than Model **A.** Clear evidence is shown that models trained with real and synthetic data (M(a,t) and M(a,v)), on balance, performed better than models trained with only-real or only-synthetic data.

Yes, there is strong evidence that training with real *and* synthetic data is better than training with either source individually. See Fig 6. In all comparisons, at least 75% of bootstrapped samples resulted in model M(a,.) having better performance (rMAE or rWIS) than the analogous model trained with either real data alone or synthetic data alone. In all comparisons presented in Fig 6, there is no evidence that training with real and synthetic data is harmful relative to training with either source individually, making this joint training the safe choice (that is, using all available training data yielded better results than a subset).

Before moving on to question Q3, it’s worth taking a moment to contemplate *why* training on synthetic data alone outperformed training on real data alone (Q1) and why training on both sources outperformed either one individually (Q2). A simple hypothesis is related to the amount of training data available for each model: there are more synthetic training data (~9 million examples) than there are real training data (~2 million examples), and there are (by definition) more real plus synthetic training data (~20 million examples) than there is of either source individually. Full training-data summaries are provided in Table 4. Under this hypothesis, more training data equates to better model performance. That is, maybe when comparing M(i,.) to M(j,.) for two different training data types i and j, the performance can be predicted simply by knowing the amount of training data available.

**Fig 7:**
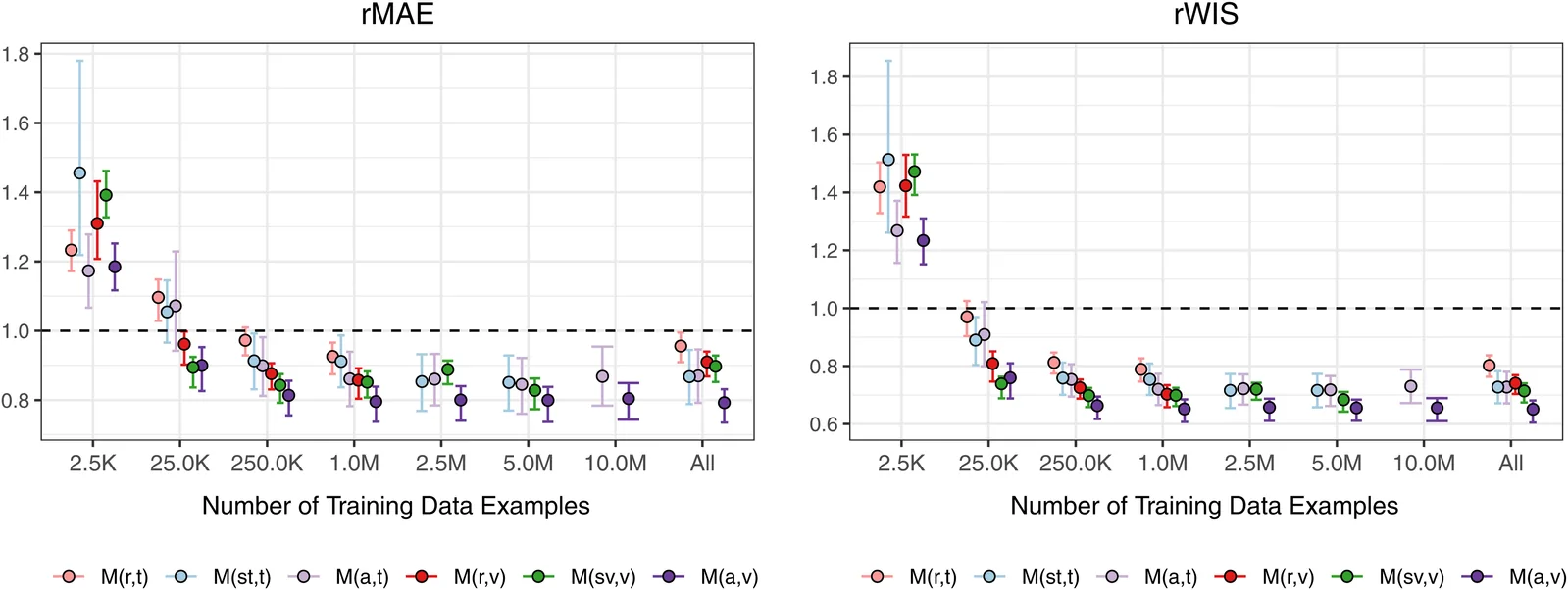
The relative mean absolute error (rMAE) and relative weighted interval score (rWIS) versus the number of training data examples for all models (sans M(st,v) and M(sv,t) — the synthetic models where the training data and the input data type are mismatched). Model performance improves as training data size increases until about one million training examples are used. “All” means the results when all available training examples for each model are used. Results presented are based on training runs with 25k model weight updates.

To investigate this hypothesis, we retrained the models on training data sizes of {2.5k, 25k, 250k, 1M, 2.5M, 5M, 10M} by taking subsets of the available training data of each type for all training data sizes less than or equal to all the available data. So, for clarity, M(r,.) is trained on training data sets of sizes 2.5k, 25k, 250k, 1M, as well as 2.1M, the full set of available real training examples. Similarly for models M(st,.), M(sv,.) and M(a,.). For each model/training data size, we calculate rMAE and rWIS averaged across all states, forecast dates, and forecast horizons. If our hypothesis is correct and the amount of training data is the driving force behind forecast improvement, we would expect to see similar performance for all models when trained on the same amount of training data, holding the input data type (TC or VAC) constant. If, however, for the same amount of training data we see some models consistently outperform other models (e.g., if M(a,.) > M(r,.)), that would be evidence against our hypothesis and would suggest the amount of training data does not solely explain why some models outperform others. Results from this exercise are shown in Fig 7.

**Fig 8:**
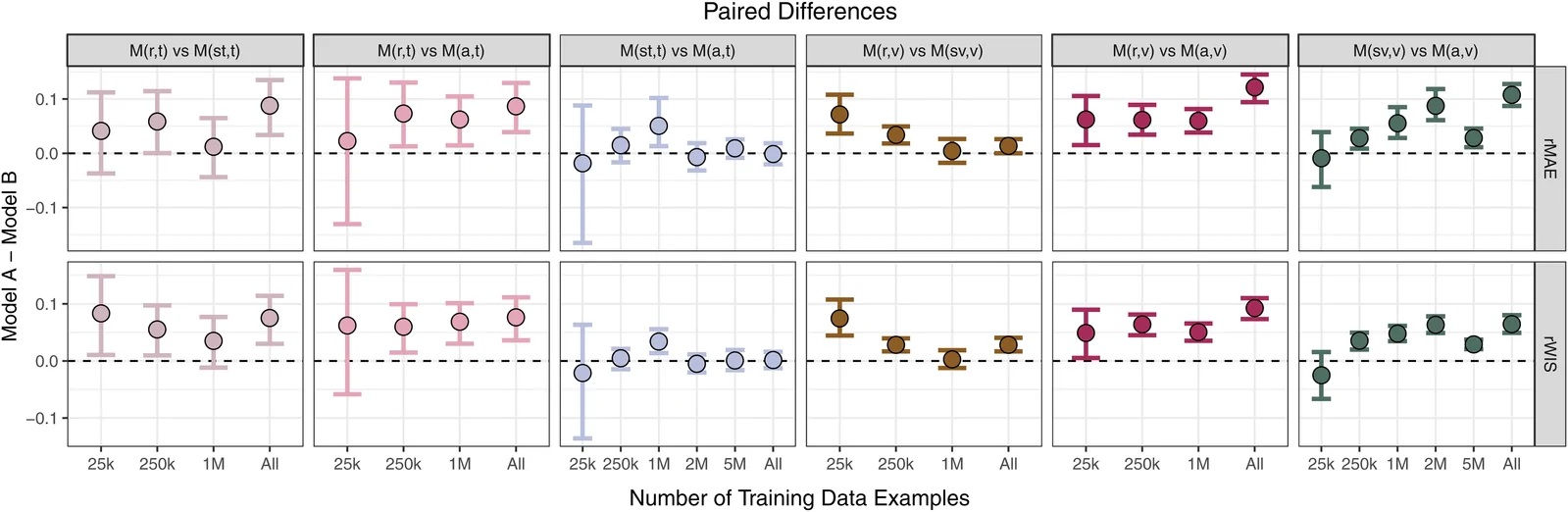
The 95% confidence intervals (bars) and averages (points) in relative mean absolute error (rMAE) and relative weighted interval score (rWIS) for paired differences of models (columns) based on 5,000 bootstrap samples. Training data sizes are shown on the x-axis. “All” means the results when all available training examples for each model are used. Results are presented as Model A - Model B, and positive values mean that Model B performed better than Model **A.** Results only shown for 25k or more training examples.

Fig 7 shows rMAE and rWIS for increasing amounts of training examples. We see that as the amount of training data increases from 2.5k examples to 1M examples, the performance of each model generally improves. In this regime, there is evidence that more training data correlates with better model performance. After models are trained with 1M training examples, however, forecast performance appears to level off. More importantly, there do appear to be systematic differences in model performance, even when holding the amount of training data constant. This can more clearly be seen in Fig 8.

Fig 8 shows the 95% confidence intervals for paired differences in rMAE and rWIS across 5,000 bootstrapped samples. While not universal, there is clear evidence shown in Fig 8 that the model trained with real and synthetic training data outperforms the models trained with only real or only synthetic training data *even after* holding the number of training data examples constant (2nd, 3rd, 5th, and 6th columns of Fig 8). Furthermore, the models trained only on synthetic data outperform those trained only on real data, even when the number of training examples is held constant (first and fourth columns of Fig 8). Fig 8 *presents evidence against our hypothesis* that the amount of training data is the explanation for M(a,.) outperforming all other models (Q2) and M(st,t)/M(sv,v) outperforming M(r,t)/M(r,v) (Q1). That is, something more than training data quantity is needed to explain the findings in Q1 and Q2.

Our other hypothesis for why training on real and synthetic data outperforms either source individually and why training with synthetic data only outperforms training with real data only centers around *covariate shift* [40]. Covariate shift in supervised machine learning (ML) refers to the change in the distribution of the input examples to a ML model between the training data (i.e., non-COVID-19, real respiratory data or synthetic data) and the testing data (i.e., COVID-19 data). Most supervised ML models assume that the training and testing data inputs (i.e., in this work, the last 20 observations of a time series) come from a common distribution. When there is a distributional mismatch, predictive performance can suffer. It could be the case that COVID-19 data are better represented by synthetic data than historical non-COVID-19, respiratory data (i.e., it could be synthetic data and COVID-19 data appear more alike than non-COVID-19, real respiratory data).

**Fig 9:**
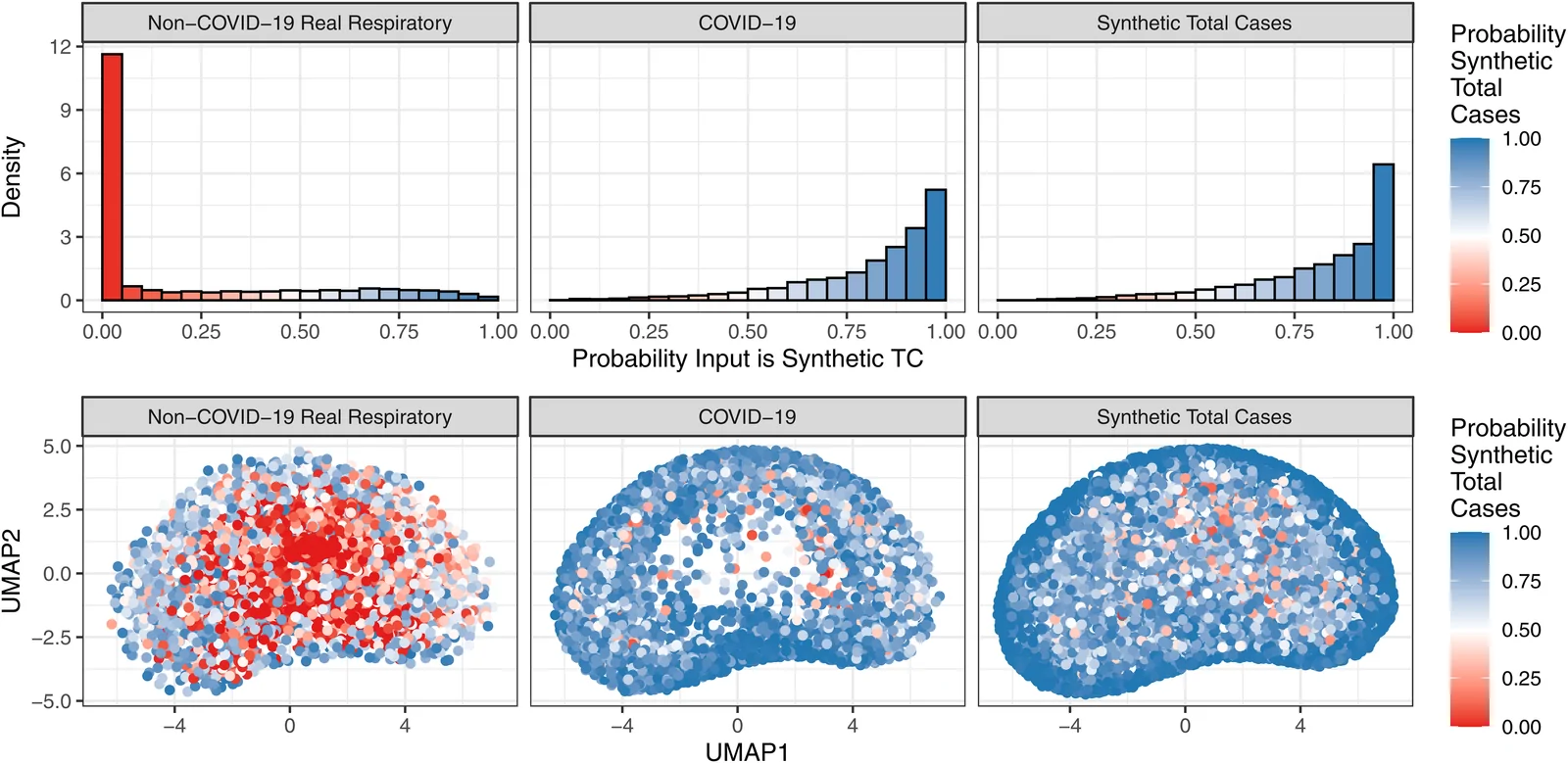
(top) The distribution of predicted probabilities that input data are synthetic (total cases) from a hold-out set. 8,000 hold-out examples from the non-COVID-19 real respiratory and synthetic total cases, respectively are being summarized along with 6,885 COVID-19 examples. COVID-19 data are overwhelmingly classified as synthetic data rather than non-COVID-19, real respiratory data, while the classifier correctly classifies synthetic data as synthetic data and non-COVID-19, real respiratory data as non-COVID-19, real respiratory data. (bottom) UMAP arrangement of hold-out data, colored by the predicted probability synthetic. We can see the non-COVID-19, real respiratory data occupies a different part of UMAP space that the COVID-19 data, while the COVID-19 data and synthetic data have more overlap in UMAP space, shedding light on why the classifier classifies COVID-19 data as synthetic.

To investigate this possibility, we fit a gradient boosted model to perform binary classification trained on 150k non-COVID-19, real respiratory training examples and 150k synthetic TC training examples. The inputs to this model were the last 20 observations of a time series (mean centered and standard deviation scaled) and the labels “Synthetic TC” or “Real” were the output. No COVID-19 data were used to train this classifier. We then ran three different hold-out data sets through the classifier: 1) 8,000 instances of non-COVID-19, real respiratory data, 2) 8,000 instances of synthetic TC data, and 3) all 6,885 instances of COVID-19 data. The non-COVID-19, real respiratory data and synthetic TC data sets were used to evaluate the classifiers capabilities. The COVID-19 data were used to see if COVID-19 data better resemble non-COVID-19, real respiratory data or synthetic TC data. If the COVID-19 data are classified as “Synthetic TC” data, that would constitute evidence that COVID-19 data come from a distribution more similar to synthetic data than non-COVID-19, real respiratory data. Results are shown in Fig 9.

Overwhelmingly, COVID-19 data were classified as synthetic data rather than non-COVID-19, real respiratory data. This is seen in the top of Fig 9. Over 90% of COVID-19 instances were assigned a probability over 0.5 that the instance was synthetic TC data. The non-COVID-19, real respiratory data and the synthetic TC data were, with high probability, correctly classified as well. Over 90% of synthetic TC instances from the hold-out set were correctly assigned a probability over 0.5 of being synthetic TC, while just under 80% of the non-COVID-19, real respiratory data were assigned a probability over 0.5 of being non-COVID-19, real respiratory data. These results indicate that the trained classifier can discriminate between real and synthetic TC data.

The bottom of Fig 9 provides a UMAP [41] (Uniform Manifold Approximation and Projection) plot — a lower dimensional representation of the 20-dimensional inputs. UMAP finds a low-dimensional embedding of high-dimensional data that preserves local neighborhood relationships. Points that are close together in the 20-dimensional space are close together in the 2-dimensional plot in Fig 9 (and points that are far away remain far away). This UMAP plot helps explain why the classifier was able to discriminate between real and synthetic data and explain why COVID-19 data was overwhelmingly classified as synthetic data. The non-COVID-19, real respiratory data largely occupies the center of the UMAP plot. The synthetic data largely covers the whole space, but is particularly concentrated around the outer ring. The COVID-19 data also largely occupies the outer ring and, notably, does not occupy the center of the UMAP shape. Thus, there is evidence that the COVID-19 data inputs better match the distribution of inputs produced by synthetic data. Or, said another way, the covariate shift between non-COVID-19, real respiratory data (training) and COVID-19 data (testing) is much more pronounced than between synthetic data (training) and COVID-19 data (testing). Because the real and synthetic data, however, occupy different regions of input space, combining them results in improved input space coverage, providing insight into why M(a,t) and M(a,v) outperformed their modeling counterparts.

#### 2.2.3 Q3a: Does training with synthetic VAC data improve COVID-19 forecasts relative to training with synthetic TC data?.

**Fig 10:**
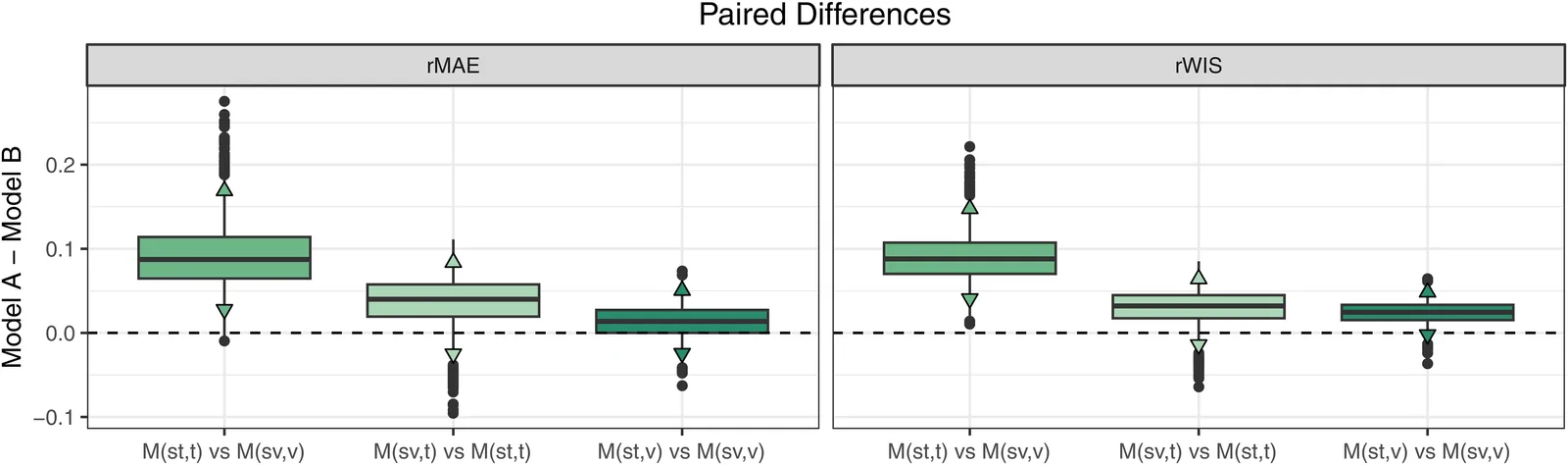
Distribution of 5,000 bootstrapped model differences for rMAE and rWIS. Triangles indicate the 2.5 and 97.5 percentiles. Presented results show Model A - Model B (on the x-axis it is displayed as M(A) vs M(B)). For instance, M(st,t) vs M(sv,v) in the rMAE panel presents the results of M(st,t)’s rMAE - M(sv,v)’s rMAE, across all bootstrap samples. Positive numbers indicate Model B performed better than Model **A.**

Yes, training with synthetic VAC data resulted in better forecasts than training with synthetic TC data. The evidence for this is shown in the M(st,t) vs M(sv,v) comparison in Fig 10. We see the 95% confidence intervals do not cover 0, indicating a statistically significant improvement in forecast performance for M(sv,v) relative to M(st,t) as measured by rMAE and rWIS.

#### 2.2.4 Q3b: Do models with matched training data and input data outperform models with mismatched training data and input data?.

Yes, there is clear evidence that models with matched training data and input data outperform models with mismatched training data and input data. This can be seen in the M(sv,t) vs M(st,t) and M(st,v) vs M(sv,v) comparisons in Fig 10. In each comparison, the model with matched training and input data (M(st,t) and M(sv,v)) outperformed their counterpart with the same input data type but different training data type in at least 75% of bootstrapped samples. This makes intuitive sense. If the model is being trained on one data type but is then being tested on a different input type, there is the potential for both covariate shift [40] (when the distribution of inputs differs between training and testing) and concept shift [42] (when the relationship between inputs and outcomes differs between training and testing).

#### 2.2.5 Q4: Does including SARS-CoV-2 variant information improve COVID-19 case forecasts?.

**Fig 11:**
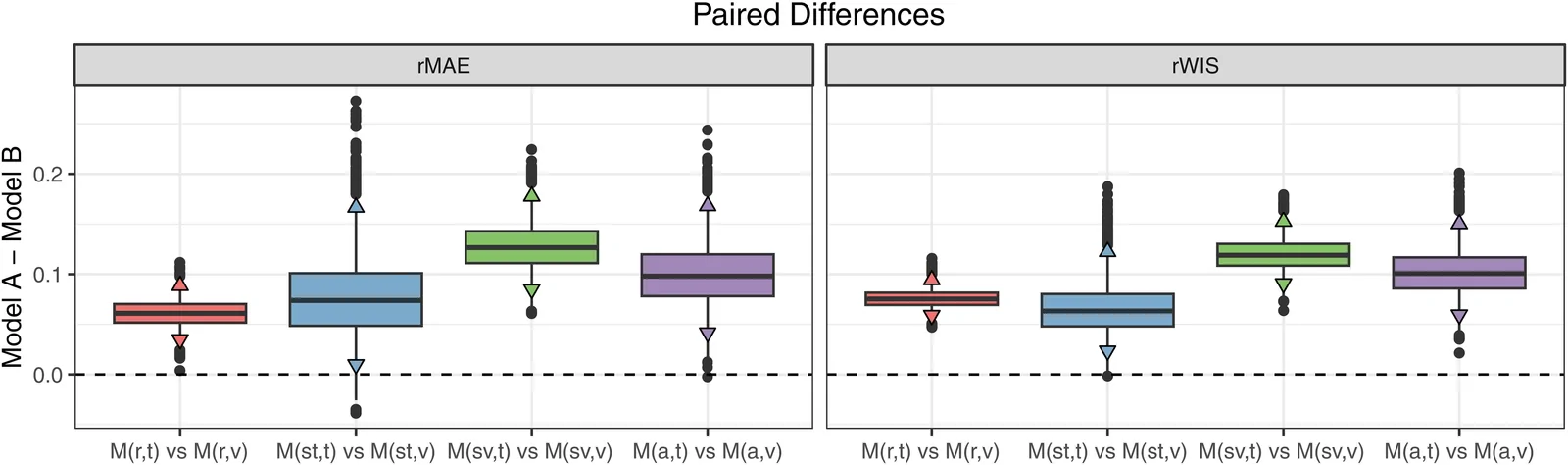
Distribution of 5,000 bootstrapped model differences for rMAE and rWIS. Triangles indicate the 2.5 and 97.5 percentiles. Presented results show Model A - Model B (on the x-axis it is displayed as M(A) vs M(B)). For instance, M(r,t) vs M(r,v) in the rMAE panel presents the results of M(r,t)’s rMAE - M(r,v)’s rMAE, across all bootstrap samples. Positive numbers indicate Model B performed better than Model **A.** Clear evidence is presented that models M(.,v) outperformed models M(.,t).

Yes, there is clear evidence that forecasting variant-attributable cases directly and summing to recover a total cases forecast improves forecasts relative to forecasting total cases directly. See Fig 11 for results. To answer this question, we compared all pairs of M(.,t) vs M(.,v). These comparisons hold the training data constant, and only differ in what data are passed in as inputs (total cases versus variant-attributable cases). The 95% confidence intervals fall above 0 for all comparisons for both metrics, indicating the models that take VACs as inputs and sum to recover total cases forecasts outperform the models that take TCs as inputs directly. Our finding that forecasting variant-attributable cases and summing produces better forecasts than forecasting total cases mirrors findings from influenza forecasting [43,44]. We conjecture that VACs have simpler and more predictable dynamics, making them easier to forecast, than the total cases time series which is a mixture of multiple co-circulating variants.

**Fig 12:**
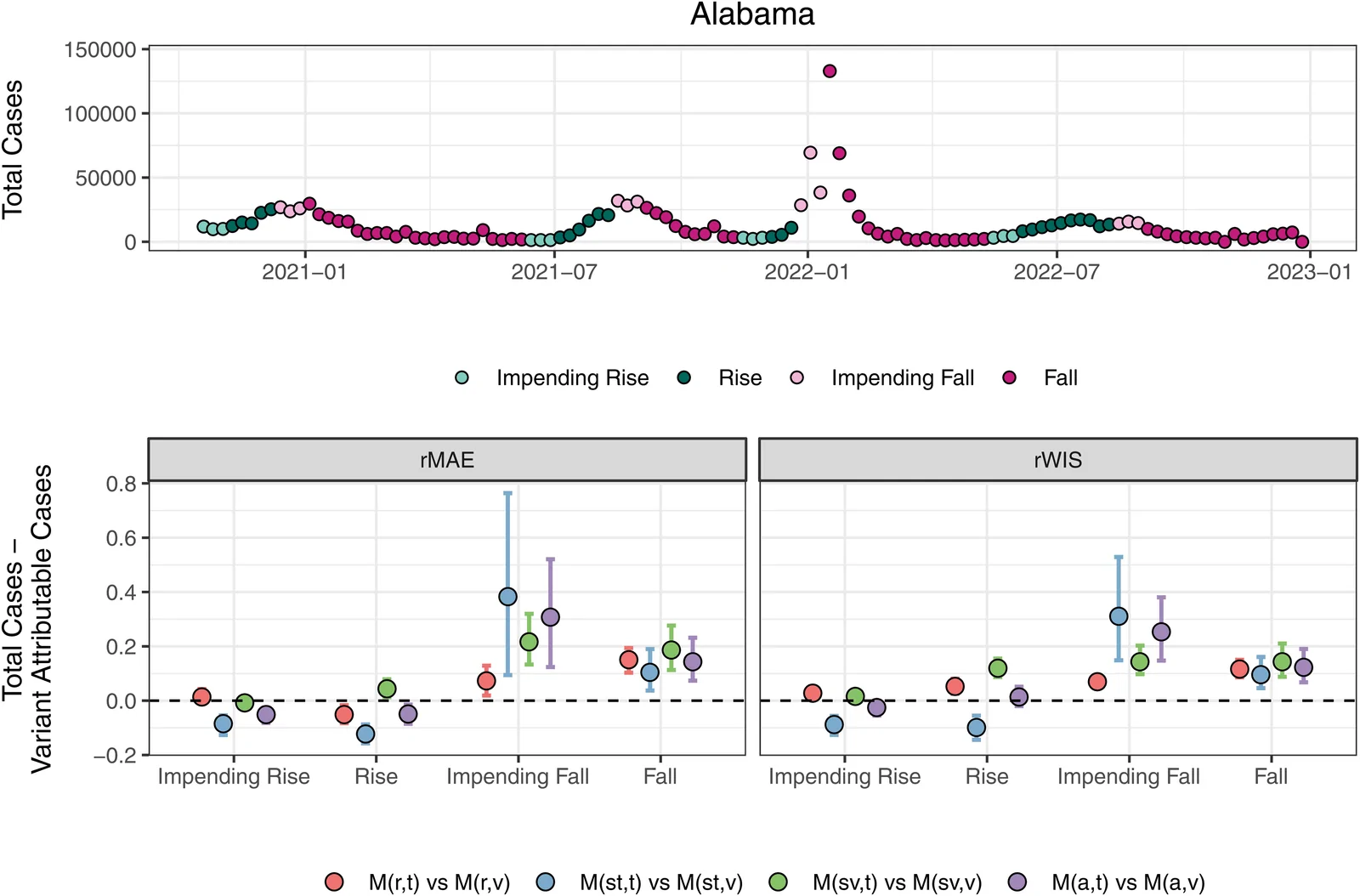
(top) Anfigu example (Alabama) of how the total cases time series is partitioned into four phases: impending rise, rise, impending fall, and fall. (bottom) The difference in rMAE and rWIS between models, broken down by phase for all states (not just Alabama). Points are average differences and error bars are 95% confidence intervals based on 5,000 bootstrap samples. Values greater than 0 mean the VAC models (M(.,v)) outperformed their corresponding TC model (M(.,t)). We see that the VAC models meaningfully outperform their TC counterparts during the impending fall and fall phases of the outbreak and perform more similarly to the TC models during the impending rise and rise phases.

Fig 12 investigates during what phases of the COVID-19 pandemic models M(.,v) outperform models M(.,t). We partition each total cases time series into four phases: impending rise, rise, impending fall, and fall. Those phases are illustrated in the top of Fig 12. We then compute the difference in rMAE and rWIS by phase for each pair of models. We see clear evidence that the M(.,v) models outperform the M(.,t) models during the impending fall and fall phases of the pandemic. VAC and TC trained models perform more similarly to one another during the impending rise and rise phases, with TC models tending to outperform VAC models when performance is measured by MAE. The degree to which the TC models outperform the VAC models during the rising phase(s), however, is small relative to the degree the VAC models outperform the TC models during the impending fall and fall phases.

#### 2.2.6 Q5: How do these forecasts compare to real-time COVID-19 case forecasts?.

To answer this question, we refer to the results presented in [1]. Evaluation in [1] included relative WIS and coverage of 1–4 week ahead forecasts from July 28th, 2020 through December 21st, 2021. This comparison should be interpreted with important context. The COVIDHub forecasts were generated in real time, using data available at the time forecasts were submitted, whereas our forecasts were generated retrospectively using finalized COVID-19 case and sequence data. Although none of our models were trained on COVID-19 observations, the synthetic-data generator and observation model were developed after the COVID-19 pandemic and may have been informed, at least indirectly, by knowledge of COVID-19 dynamics. Thus, this comparison is not a strict real-time head-to-head evaluation, but rather a benchmark against a strong real-time ensemble over a common evaluation period.

**Fig 13:**
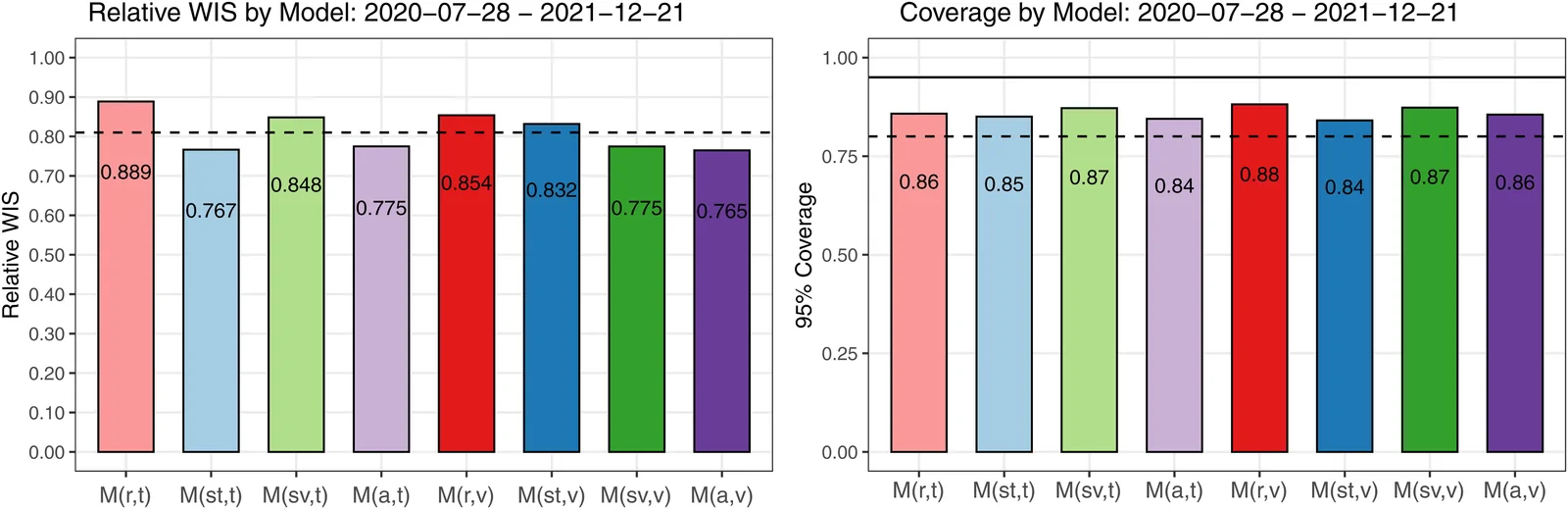
(left) Relative weighted interval score (rWIS) corresponding to the evaluation period of July 28th, 2020 through December 21st, 2021. Lower is better. The COVIDHub-4_week_ensemble had a relative WIS of 0.81 [1] (the horizontal dashed line). Models with rWIS below the horizontal, dashed line outperformed the COVIDHub-4_week_ensemble as measured by rWIS. (right) Empirical coverages for a 95% nominal prediction interval (solid line). The COVIDHub-4_week_ensemble’s 95% prediction intervals had an empirical coverage of 0.8 (dashed line). All models had empirical coverages closer to nominal than that.

See Fig 13 for WIS and coverage results for all models restricted to July, 2020 through December, 2021. All eight models outperformed a persistence (baseline) model (a feat only 7 out of 22 models accomplished in real-time). In this retrospective comparison, four models had lower rWIS than the COVIDHub-4_week_ensemble model, which had a relative WIS of 0.81 [1]. These models were the models trained on synthetic data only where the training data type matched the input data type (M(st,t) and M(sv,v)) as well as the two models trained with all the available training data (M(a,t) and M(a,v)). The models that did not outperform the COVIDHub-4_week_ensemble model were the models trained exclusively on real training data (M(r,t) and M(r,v)) and the models trained exclusively on synthetic data with a mismatch between training data type and model input type (M(sv,t) and M(st,v)). For additional context, even the worst performing model considered in this paper, M(r,t), had an rWIS that would rank fifth among the real-time forecasting models summarized in [1], again, recognizing the retrospective nature of our evaluation. Furthermore, the COVIDHub-4_week_ensemble had a 95% coverage of 0.8. All eight models had an empirical coverage between 0.84 and 0.88, closer to nominal than did the COVIDHub-4_week_ensemble. Additionally, although the VAC data input models (M(.,v)) could not have been forecast in real-time due to variant reporting delays, the TC data input models (M(.,t)) could have been forecast in real-time. It is also worth a reminder that none of the eight models in this paper used any COVID-19 data for training; they only used data (real and/or synthetic) that would have been available on or before January 1st, 2020.

## 3 Discussion

In this paper, we sought to answer two high-level questions: (1) Can synthetic data be used to train deep learning models that achieve state-of-the-art infectious disease forecasting performance? and (2) Can genetic information be used to improve infectious disease forecasting models? We conducted an exercise to answer these questions with an eye towards the next pandemic by limiting ourselves to only using training data available prior to January 1st, 2020 for forecasting COVID-19 cases. We affirmatively answered both questions. Synthetic data was a valuable training data source, as models trained on only synthetic data provided impressive forecasting performance. Real historical data for non-COVID-19 pathogens also proved to be a valuable training data source, though less valuable than synthetic on its own (Q1). Combining real and synthetic data was found to be a prudent path forward, as models trained with all available training data outperformed models trained on real or synthetic data alone (Q2). Furthermore, forecasting variant-attributable cases directly and summing of forecasts produces demonstrably better forecasts than forecasting total cases directly (Q4). Model M(a,v), the model that made use of all available training data and used variant information, was the best performing model.

The deep learning models trained and used in this exercise required training once, but required no retraining. While deep learning models can take several hours to train (details in Table 4), they take seconds to predict with and can scale to a large number of forecast settings. While we trained our models once, we could have either retrained every week as new data became available, or, more commonly, performed fine-tuning on our pre-trained model with the new COVID-19 data as it became available (e.g., [45]). Thus scaling a forecasting model from, say, US states to all US counties would be straightforward. This is in contrast to a spatial or hierarchical model that borrows information across locations, where retraining may be required every time new data are made available and scaling up from tens to hundreds or thousands of locations is not trivial (e.g., [3]). Again, these deep learning models require adequate amounts of training data to be effective, training data that are not available in an emerging disease setting. Historical outbreak data of related disease and/or synthetic data proved to be useful training data. This paper adds to the growing body of evidence that synthetic data is a valuable and under-tapped resource for infectious disease forecasting [36–38], as it provides the necessary ingredient to unlock the potential of deep learning models.

There are many opportunities to push this deep learning plus synthetic data idea further. Going back to the input/output deep learning framework, many future forecasting opportunities for improvement amount to augmenting the inputs. Forecasting geographic locations jointly rather than one at a time is an exciting path forward (recent real-time influenza forecasting success has employed a hierarchical modeling approach that borrows information across locations [3,46]). This amounts to developing training examples where, say, recent observations from all US states are the input and the next H time steps for each US state is the output, allowing the deep learning model to learn (potentially) complex inter-state relationships. To learn these relationships, however, will require suitable training data. MutAntiGen has multi-location capabilities thus could be a suitable synthetic data generator. Another application is jointly forecasting cases, hospitalizations, and deaths (as was done in [36]). Rather than forecasting variant-attributable cases, one could consider computing population genetic summaries of the viral population as time series and providing those as inputs paired with total cases. This approach would require a synthetic data simulator with appropriate evolutionary biology fidelity. In general, for this deep learning plus synthetic data idea to scale will require simulators sophisticated enough to make training data with sufficient realism, which will likely require continued simulator development.

While synthetic data is a promising resource to use, it is not a panacea. How to generate it requires thoughtful contemplation of the problem at hand. For instance, for much of the COVID-19 pandemic, data was collected and disseminated in the US at daily resolution, introducing day-of-week effects. Such effects are learnable and exploitable, but for a deep learning model to incorporate them, it would need to be presented with training data reflecting those patterns. We did not create any synthetic data having day-of-week effects, so we anticipate our approach might fail if applied to daily data. That said, to forecast at the daily scale, we could add a day-of-week routine to the observation model described in S1 Text. Similarly, to use this approach to forecast seasonal diseases, we would want to both ensure we are synthetically generating seasonal outbreaks as well to increase the context window (up to, say, C = 52 or 104), capturing at least one entire period of the outbreak.

Finally, while we conducted this retrospective forecasting study with an eye towards real-time forecasting, we acknowledge that reporting delays exist with both epidemiological and genetic data. Our results represent best-case scenarios with respect to reporting delays and more work is needed to bridge the real-time reporting delay gap.

## 4 Materials and methods

This section provides details on the study design, data sources, synthetic data generation process, forecasting model, and evaluation metrics used in the results above.

### 4.1 COVID-19 study details and data overview

#### 4.1.1 Scope of study.

The study details are presented in Table 2. COVID-19 cases were selected as the target because they serve as a leading indicator of more severe outcomes like hospitalizations and deaths and were empirically difficult to forecast [1]. The time range and cadence correspond to data availability and public health relevance. U.S. states (plus Puerto Rico) were selected as a geographically relevant unit for public health. There is a large volume of SARS-CoV-2 genomes for US states between June 2020 and December 2022, allowing us to test the value of genetic information. Furthermore, major COVID-19 data collection and dissemination resources ramped down operation in March of 2023 [47].

**Table 2:** Study details, including the evaluation metrics of mean absolute error (MAE) and weighted interval score (WIS).

Category	Details
Disease and Target	COVID-19, Cases
Time Range	June 2020 – December 2022
Time Cadence	Weekly
Geographic Extent	USA
Geographic Resolution	States/Territories
Forecast Horizon	1–4 weeks ahead
Evaluation Metrics	MAE, WIS, and coverage

#### 4.1.2 Real COVID-19 case data.

**Fig 14:**
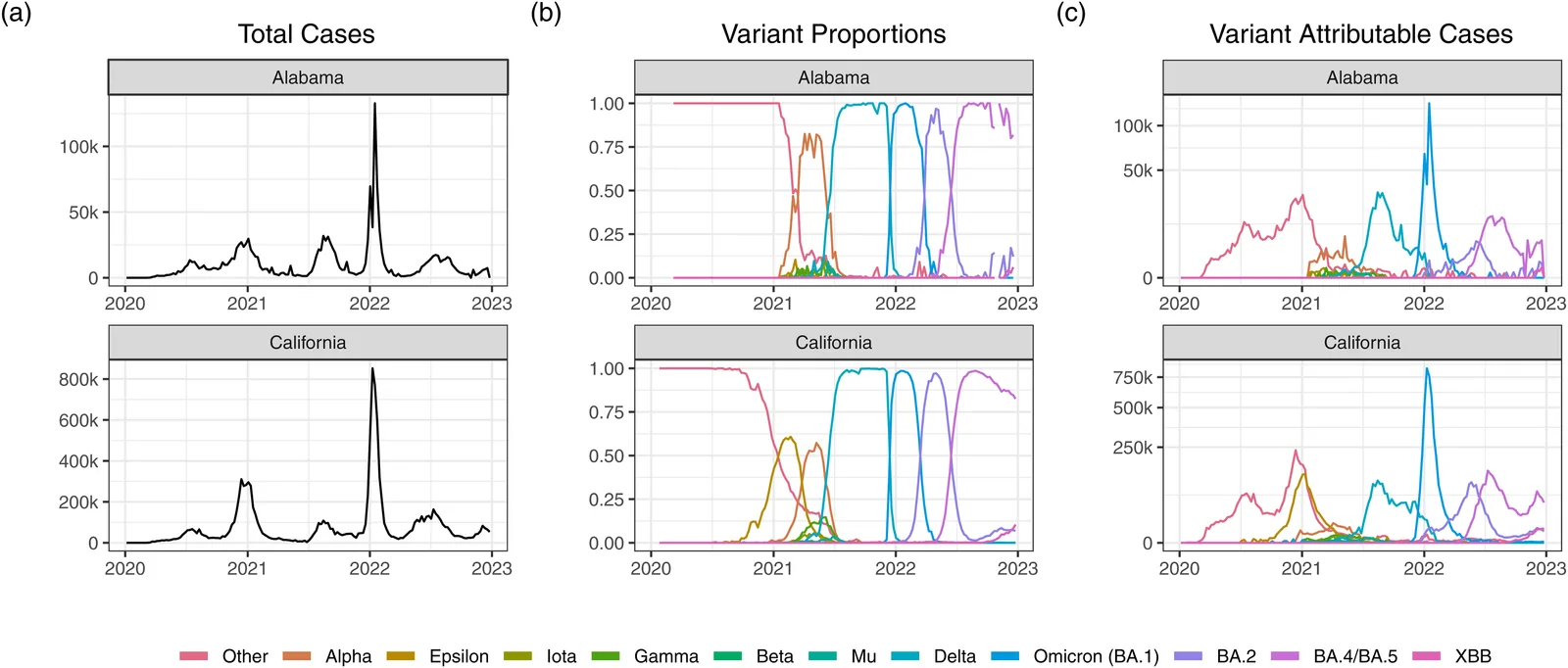
COVID-19 data for Alabama and California. **(a)** Weekly total cases (TCs). **(b)** Proportion of sampled viral genomes assigned to each variant. **(c)** Variant-attributable cases (VACs), computed as TCs times the proportion of genomes assigned to each variant. VACs summed over all variants equal the TCs. Note the square-root scale on the y-axis for better visibility of low-count VACs.

Data on COVID-19 cases between December, 2019 and March, 2023 were obtained from the Johns Hopkins University Center for Systems Science and Engineering (CSSE) GitHub (https://github.com/CSSEGISandData/COVID-19_Unified-Dataset) [48]. This database includes COVID-19 case data compiled from a variety of sources; our analysis prioritized case counts for each location and date as reported from large, curated, cross-national databases when available. Delays in real-time reporting of COVID-19 cases were ignored in this analysis, and the data reported as of September 2023 (the date the data were pulled) for a given date were treated as known as of that date. As examples, we show the weekly total COVID-19 case counts for Alabama and California over the study period (Fig 14).

#### 4.1.3 Real COVID-19 genetic data.

The COVID-19 pandemic generated an unprecedented global collection of viral genome sequences, shared through repositories and data-sharing platforms including GISAID [17], GenBank [49], the European Nucleotide Archive [50], and, more recently, Pathoplexus [51]. Of these sources, GISAID provided the most sequence records, the broadest global representation, and the shortest times between sample collection and data release [18]. In this paper, we made use of approximately 4.5 million GISAID records for samples collected in the United States between June 1st, 2020 and December 31st, 2022. Rather than using raw genomic sequences, in this work we group sequence variants by Pango lineage designation [52] as assigned in the GISAID metadata as of September 2023; each genome is assigned to one of many discrete variant categories, which we aggregate into coherent encompassing supergroups. Aggregating these labels across time and location yields variant proportion time series, such as those shown in Fig 14B. We combine variant proportion time series with total cases time series to compute variant attributable case (VAC) time series (e.g., Fig 14C), where variant-attributable cases for each time point equal total cases times variant proportion.

We note that there is a meaningful delay between when a viral sample is collected and when its genome and metadata become publicly available. That lag — driven by lab turnaround, quality control, metadata completion, and curation — varies across geography and time [53]. This paper, like many retrospective studies, neglects this delay, but operational forecasting would need to model it. As such, the results in this paper for the models that use VACs as their input time series should be viewed in their appropriate context.

### 4.2 Training data

#### 4.2.1 Real, non-COVID-19 respiratory disease data.

As early as late 2019, the outbreak later attributed to SARS-CoV-2 was described clinically as an acute respiratory illness (pneumonia) [54]. While little to no COVID-19 data would have been available on January 1st, 2020, we could have known that COVID-19 produced symptoms consistent with respiratory diseases. For this reason, we use non-COVID-19, real respiratory data available prior to January 1st, 2020 for training in this paper. All data and code used in this paper are available at https://github.com/lanl/precog/tree/main/synthetic_and_genetic_forecasting, originally derived from data found at https://github.com/lanl/precog/tree/main/infectious_timeseries_repo. Non-COVID-19 respiratory diseases include influenza, pneumonia, mumps, RSV, tuberculosis, and diphtheria (among others). In this paper, we will often use the shorthand “real data” or “real training data” to mean “non-COVID-19, real respiratory disease data.” For example, the “Training Data = Real” in Table 1 means “non-COVID-19, real respiratory disease data.”

**Fig 15:**
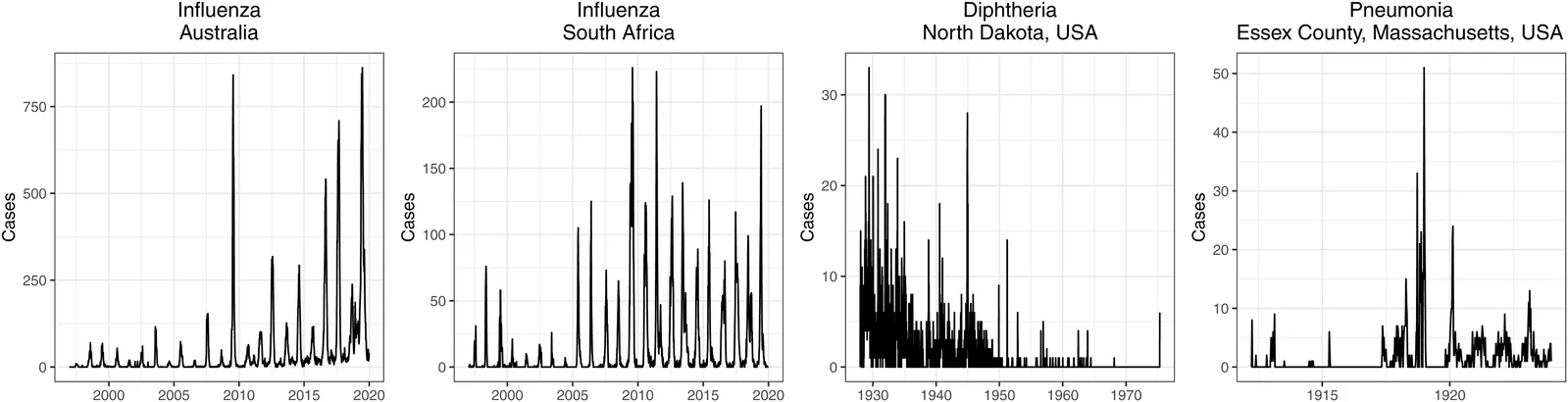
Examples of non-COVID-19, real respiratory data. Over 2,000 time series are available for training, amounting to over 2 million observations.

As can be seen in Table 3, about 2,000 non-COVID-19, real respiratory time series are available for training. Those time series have an average length of about 1000 observations, and a median length of about 550 observations. A selection of the non-COVID-19, real respiratory time series are shown in Fig 15.

**Table 3:** Training time series summaries. “Real” means “non-COVID-19, real respiratory,” “TC” means “Total Cases,” and “VAC” means “Variant-Attributable Cases”.

Training Data	# of Time Series	# of Total Obs.	Avg. (Median) Time Series Length
Real	2,167	2,169,760	1001 (551)
Synthetic, TC	36,600	9,460,880	258 (256)
Synthetic, VAC	36,570	10,664,618	292 (294)

#### 4.2.2 Synthetic data.

In addition to the real data, we generated synthetic data to represent a wide range of *possible* disease behaviors. The intent of these synthetic data is to discover behaviors that are within scope of possible disease dynamics, yet not explicitly represented in the available real data observations. We generate synthetic disease data via the MutAntiGen agent-based model (ABM) [39,55,56] because it can generate viral variant turnover dynamics.

**MutAntiGen.** In agent-based modeling, a large-scale ecological system is simulated as a collection of autonomous decision-making entities called agents [57]. In the MutAntiGen ABM, originally developed [39] as an extension of an antecedent ABM [55], “agents” are categorized as either infected or non-infected individuals in a population susceptible to disease spread. MutAntiGen explicitly models the joint behavior of an evolving pathogen and dynamic susceptibility/resistance of the host population, allowing for non-seasonal case waves. Further details on MutAntiGen are available in S1 Text.

**Fig 16:**
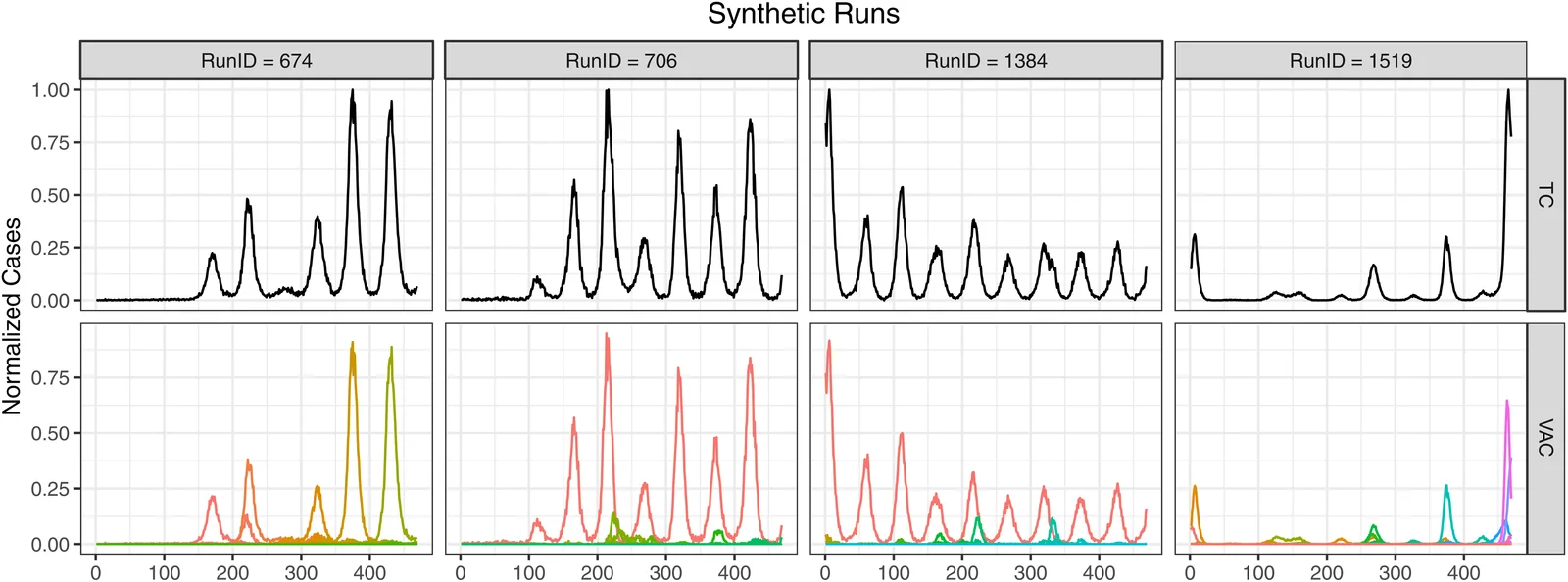
MutAntiGen example runs. MutAntiGen outputs both the total number of cases (top row, TC) and the time series of cases attributed to each variant (bottom row, VAC; each line and color represents a different variant). For each time point, the sum of all variant-attributable cases (bottom row) equals the total cases (top row).

Some example runs of MutAntiGen are shown in Fig 16. In addition to cases over time, the simulator reports viral samples from a subset of infections. This yields time series of cases attributable to antigenic types, as shown in the lower panels of Fig 16. Viral sampling typically is proportional to number of cases, but this yields very few samples when cases are low before a new wave begins, which is exactly when data are critical for a forecasting model. We therefore modified the MutAntiGen code to sample with greater intensity when cases are low.

As one might imagine, an ABM intended to represent a massively complicated environmental system includes many parameters to be set by the user prior to running a simulation. These parameters greatly affect the outcomes of the simulation, but it is still difficult to predict the results of the simulation due to the innate stochasticity of ABMs [58]. The required MutAntiGen parameters and their biological interpretations, as well as our computational modifications to the original code that allow for better sampling at scale, are discussed in S1 Text.

**Design to produce MutAntiGen runs** Our goal in generating synthetic data was to produce a rich and diverse set of training data for a forecasting model, emphasizing effective representation of both antigenic mutation (an individual property) and turnover in dominant antigenic type (a property of populations). Since the aim is to forecast emergent diseases, we are unlikely to know ahead of time what parameter choices will best reflect an impending outbreak; therefore these simulations must cast as wide a net as is practicable, including a broad range of model parameters so that future outbreaks would be more likely to fall within that net (i.e., be represented in the sample space).

To select the parameter values we run MutAntiGen at, we draw a Latin hypercube sample (LHS) [59]. Our default MutAntiGen parameter values and ranges were informed by the literature and calibrated to represent global influenza H3N2 phylodynamics. We expanded a subset of the parameters that control the evolutionary, epidemiological, and immunological dynamics to yield more general simulations. For parameters with established empirical estimates, we set plausible bounds based on data from representative RNA viruses including influenza A, influenza B, Measles, Nipah, Dengue, Zika, and Hepatitis C.

By nature of LHS’s independent sampling, many combinations of unrealistic or unknown viruses could be generated, with parameter combinations that do not respect known biological constraints (e.g., error catastrophe, mutation scaling rates [60,61]) or outbreak conditions (*R*_0_ > 1). However, we allowed such combinations under the assumption that biologically nonviable regimes would result in simulation failure or additional data that the model would be able to determine to be irrelevant for the prediction task. Previous work has shown that the risk of negative transfer, where model performance degrades with the addition of new data, is low as long as at least some of the training data resemble the disease being forecasted [62]. The specifics of parameter bounds used in these simulations, our rationales for selecting them, and a full list of MutAntiGen parameters held constant are available in S1 Text.

**Fig 17:**
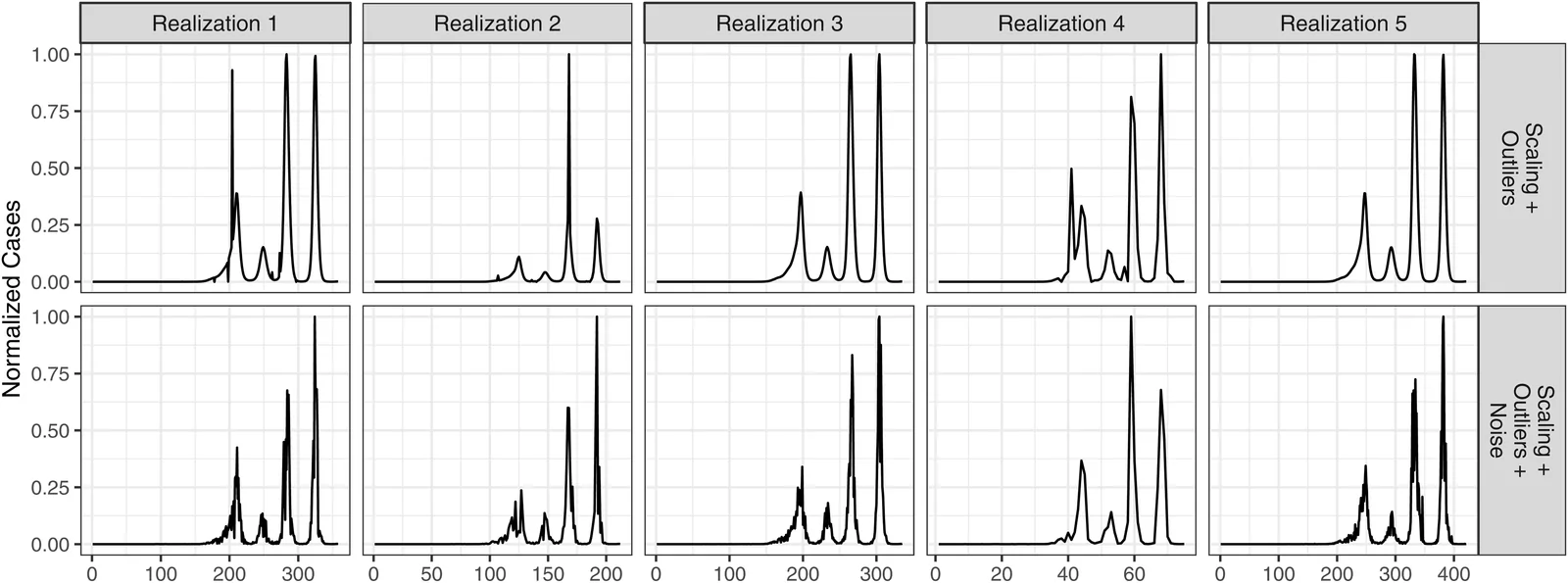
10 of the 20 realizations from the observation model corresponding to a single MutAntiGen output. Realizations were generated by subjecting the “clean” MutAntiGen output to either scaling (random-magnitude compression of the x-axis) and (possible) addition of outliers (top row), or to scaling plus addition of noise and (possibly) outliers (bottom row).

**Observation model for synthetic data** As can be seen in Fig 16, the output of MutAntiGen can have unrealistically low noise. Real data, in contrast, are noisy, biased, and often include outliers (compare Fig 15 to Fig 16). That is, real data can be thought of as imperfect versions of idealized epidemiological data. In an effort to make the synthetic outputs of MutAntiGen more realistic, we passed each MutAntiGen output through an observation model 20 times, resulting in different imperfect versions of each MutAntiGen time series. At a high level, the observation model applies three possible transformations. First, it rescales the time axis by linearly interpolating each simulated outbreak to a shorter time series, allowing the same simulated dynamics to occur over faster timescales. Second, for half of the observation-model realizations, it applies multiplicative noise to the case counts. Third, each realization has a probability of receiving both high and low outliers, representing reporting artifacts such as data dumps or missed reports. This observation model increases both the amount and the diversity of the training data. Fig 17 shows different realizations of the observation model for a single MutAntiGen output. After sending all MutAntiGen runs through the observation model process, we generated approximately 36,000 total time series for training for both the total cases and the variant-attributable cases (see Table 3 for specifics). More details of the observation model can be found in S1 Text.

### 4.3 Forecasting model

#### 4.3.1 Training.

We frame our probabilistic forecasting problem as a conditional quantile regression problem. Let


$$\begin{array}{c} y_{t+h}|{𝐲}_{t}^{in}\sim F_{{𝐲}_{t}^{in},h} \end{array}$$ (1)

be the conditional distribution for $y_{t+h}$, the number of new infections reported at time *t* + *h* for t∈{1,2,...} and h∈{1,2,...,H} given the last *C* newly reported infec*t*ions ${𝐲}_{t}^{in}=y_{(t-C+1):t}$. In this paper, we set *C* = 20 and *H* = 4. The context length *C* = 20 was chosen as a practical compromise rather than through a formal optimization study. Shorter context windows allow the model to be used earlier in an outbreak, since at least *C* observations are needed before a forecast can be generated. Longer context windows provide more recent history from which the model can infer outbreak dynamics, variant turnover, and changes in trajectory. We chose 20 weeks to provide several months of recent context while still allowing forecasts relatively early in an emerging-outbreak setting.

We define the quantile function as follows:


$$\begin{array}{c} q_{\tau ,h}({𝐲}_{t}^{in}):=F_{{𝐲}_{t}^{in},h}^{-1}(\tau ) \end{array}$$ (2)

for τ∈[0,1]. That is, the conditional quantile function $q_{\tau ,h}()$ — doubly indexed by the quantile level τ and the step ahead *h* — is the inverse of the conditional cumulative distribution function evaluated at the quantile level τ, $F_{{𝐲}_{t}^{in},h}^{-1}(\tau )$. We approximate the inverse of the conditional cumulative distribution function $F_{{𝐲}_{t}^{in},h}^{-1}$ with a set of quantile functions $q_{\tau ,h}({𝐲}_{t}^{in})$ evaluated over a grid of quantile levels τ∈T where


$$\begin{array}{c} T=\{0.0005,0.005,0.01,0.025,0.05,0.1,...,0.9,0.95,0.975,0.99,0.995,0.9995\} \end{array}$$

and |T|=27. T constitutes a dense grid of quantile levels, denser than we use for evaluation (see Section 4.4 for details). This dense grid, however, allows us to better approximate the tails of the forecast distributions which will be needed in the forecasting of models M(r,v), M(st,v), M(sv,v), and M(a,v) (see Section 4.3.2 for details). The quantile function provides a point forecast and forecast intervals. For instance, the point forecast is the evaluated quantile function when τ=0.5 (the median). The 95% forecast interval lower and upper bounds are the quantile functions evaluated for quantile levels τ=0.025 and τ=0.975, respectively.

Given our problem statement, our next task is to estimate $q_{\tau ,h}({𝐲}_{t}^{in})$. To do that, we turn to deep learning [63]. Deep learning models are flexible function approximators. Provided an adequate amount of training data, deep learning models can learn continuous functions to high degrees of precision [64]. Using the training data described in Section 4.2, we train a 2-layer transformer model [65] that takes the last 20 observations of a time series as input and predicts the quantile levels in T for h∈{1,2,...,H}. Pinball loss is used to perform the quantile regression. Training and deep learning model details can be found in S1 Text.

The results of the model fitting are four different trained deep learning models, differing only in the training data used to learn the model parameters: real data only (i.e., all non-COVID-19, real respiratory data detailed in Section 4.2.1), synthetic total cases, synthetic variant-attributable cases, and all training data (i.e., non-COVID-19, real respiratory data, synthetic total cases data, and synthetic variant-attributable cases). As is detailed in Table 4, with *C* = 20, we are able to generate between 2 and 21 million input/output pairs of training data where ${𝐲}_{t}^{in}$ is the input and ${𝐲}_{t}^{out}=y_{\left(t+1):(t+H\right)}$ is the output. To be clear, if there is a training time series of length *T* = 100, that will produce 100−20−4+1=77 input/output training data examples (e.g., $\left\{{𝐲}_{C}^{\text{in}},{𝐲}_{C}^{\text{out}}\right\}$, $\left\{{𝐲}_{C+1}^{\text{in}},{𝐲}_{C+1}^{\text{out}}\right\}$, ..., $\left\{{𝐲}_{T-C-H+1}^{\text{in}},{𝐲}_{T-C-H+1}^{\text{out}}\right\}$).

**Table 4:** Training details for each training data set. Each model was presented with approximately 25 million training examples during learning. Example views is the average number of times each training example was viewed by the model during training (total number of training examples viewed by the model divided by the total number of unique available training examples). The closer example views is to 1 (or less), the less likely the model is to memorize the training data and thus the less likely the model is to overfit the training data. Training time does not scale with the number of training examples but rather the number of training examples presented to the model during training, which was held fixed at approximately 25 million for all models. Training times presented here used a 2023 MacBook Pro with Apple M2 Max and 64 GB RAM. Model training was CPU-only and did not use GPU acceleration. Code was run in R 4.4.3 with models fit using the torch package version 0.16.3.

Training	Number of Training	Example	Training Time
Data	Examples (millions)	Views	(minutes)
Real	2.1	11.8	706
Synthetic, TC	8.6	2.9	701
Synthetic, VAC	9.8	2.5	701
All	20.6	1.2	702

Each fitted model was trained for a fixed run length of approximately 25 million presented training examples. This value was chosen as a practical, conservative training length rather than through a formal tuning exercise. We used the same run length for all training data sources so that differences across models would not be driven by different numbers of weight-update opportunities. During training, validation pinball loss was monitored, and the final forecasting model was selected as the exponential-moving-average checkpoint with the lowest validation loss.

Table 4 presents summaries of model training. Each model was trained on between 2 and 21 million unique training examples. Each model was presented with approximately 25 million training examples during training. Thus, each training example was viewed by the model between 1 and 12 times (example views), depending on the number of available training examples. Deep learning models run the risk of memorizing the training data and thus overfitting if they are presented the same training examples repeatedly. The large numbers of training examples shown in Table 4 help protect against overfitting. The training time is a function of the number of training examples presented to the model (25 million), not the number of available training examples. This is why the training time does not scale with the number of training examples. The time to train the model (almost 12 hours) would be considered expensive (possibly prohibitive) if retraining was required every week when new data become available for real-time forecasting. The forecasting approach considered here falls under the “expensive to train but cheap to deploy” paradigm. While it takes on the order of half a day to train these models, they only need to be trained once. Forecasting with these trained models is measured on the scale of seconds (or less), making them appealing in operational settings. It is worth noting that the training time could be shortened by making use of graphical processing units (GPUs).

#### 4.3.2 Forecasting.

Forecasting differs depending on the model input (recall Table 1). The models that take total cases as the input are straightforward to forecast. The fitted transformers produce forecasts for all quantile levels in T. We only use seven of those quantile levels, τ∈{0.025,0.1,0.25,0.5,0.75,0.9,0.975}, allowing us to produce a point forecast (the median) and three prediction intervals: 50%, 80% and 95%. These quantile predictions allow us to evaluate forecasts with respect to multiple popular metrics (see Section 4.4 for details).

Forecasting the models where the model input are the variant-attributable cases (VACs) requires one more step. For a given state, forecast date (*t*), and forecast horizon (*h*), we forecast each VAC at the dense grid of 27 quantile levels in T. Using those 27 quantiles as an estimate of the cumulative distribution function (CDF) for each VAC, we draw a realization from each VAC’s CDFs by inverting the CDF [66] and linearly interpolating between quantile estimates (this is why T has quantile estimates so far out in the tails). Given a draw from each VAC’s forecast distribution, we sum those draws constituting a single draw from the total cases forecast distribution. We repeat this sampling process N = 100,000 times. Then, we compute the same seven quantile levels {0.025, 0.1, 0.25, 0.5, 0.75, 0.9, 0.975} as the sample quantiles of the N draws from the total cases forecast distribution, derived from each individual VAC forecast distribution.

Notice that pairs of models in Table 1 use the same fitted transformer model to produce forecasts. For example, models M(r,t) and M(r,v) each use the same fitted transformer model, trained with only non-COVID-19, real respiratory data, but will yield different forecasts because the inputs to the model are different (recall Fig 14A & 14C).

### 4.4 Evaluation metrics

We evaluated forecasts using mean absolute error (MAE), weighted interval score (WIS), and empirical coverage.

MAE is defined as


$$\begin{array}{c} \text{MAE}=\frac{1}{N}\sum_{i=1}^{N}|{\hat{y}}_{i}-y_{i}| \end{array}$$ (3)

where *N* is the number of forecasts, $y_{i}$ is the observation, and ${\hat{y}}_{i}$ is the point forecast. MAE is greater than or equal to 0 and is negatively oriented (smaller is better). The point forecast $\hat{y}$ for this work is the median forecast from the quantile regression. Most of the evaluation results are presented relative to a persistence model — a model whose forecast ${\hat{y}}_{t+h}=y_{t}$ for any h≥1 (i.e., M(0)). As such, we also define relative MAE (rMAE) as follows:


$$\begin{array}{c} \text{rMAE}({\text{M}}_{i})=\frac{\text{MAE for }{\text{M}}_{i}}{\text{MAE for M(0)}} \end{array}$$ (4)

where ${\text{M}}_{i}$ is any model *i* listed in Table 1. rMAE is greater than or equal to 0 and negatively oriented. rMAE(${\text{M}}_{i}$) < 1 indicates that model ${\text{M}}_{i}$ has a better MAE than a persistence model.

WIS is a way to evaluate forecasts in an interval format. Intuitively, WIS penalizes two things: interval widths (the wider the interval width, the larger the penalty) and observations that fall outside the forecast interval (the further the observation falls outside the forecast interval, the larger the penalty). As such, WIS encourages forecasts to be sharp and well-calibrated [67]. We consider *K* = 3 central (1−α)×100% forecast intervals: 50%, 80% and 95% (corresponding to α=0.5,0.2, and 0.05, respectively). Following [68], WIS is defined as


$$\begin{array}{c} \text{WIS}=\frac{1}{K+0.5}\times (w_{0}\times |y-q_{0.5}|+\sum_{k=1}^{K}(w_{k}\times {\text{IS}}_{\alpha_{k}})) \end{array}$$ (5)

where *w*_0_ = 0.5, $w_{k}=\alpha_{k}/2$, $q_{0.5}=\hat{y}$ (the median forecast), *y* is the observations, and ${\text{IS}}_{\alpha_{k}}$ is the interval score corresponding to $\alpha_{k}$, defined as


$$\begin{array}{c} {\text{IS}}_{\alpha_{k}}=(u_{\alpha_{k}}-l_{\alpha_{k}})+\frac{2}{\alpha_{k}}((l_{\alpha_{k}}-y)*\text{I}(y<l_{\alpha_{k}})+(y-u_{\alpha_{k}})*\text{I}(y>u_{\alpha_{k}})) \end{array}$$ (6)

where $l_{\alpha_{k}}$ and $u_{\alpha_{k}}$ are the $\alpha_{k}/2$ and $1-\alpha_{k}/2$ quantiles and **I**() is an indicator function equal to 1 if the argument is true and 0 otherwise. WIS is greater than or equal to zero and is negatively oriented. Similar to MAE, relative WIS (rWIS) is defined as follows:


$$\begin{array}{c} \text{rWIS}({\text{M}}_{i})=\frac{\text{WIS for }{\text{M}}_{i}}{\text{WIS for M(0)}} \end{array}$$ (7)

where ${\text{M}}_{i}$ is model *i*. rWIS(${\text{M}}_{i}$) < 1 means model ${\text{M}}_{i}$ has a better WIS than a persistence model. The persistence model (${\hat{y}}_{t+h}=y_{t}$) does not intrinsically produce forecast intervals. We constructed prediction intervals for the persistence model following the COVIDHub-baseline procedure described in the Supplemental Materials of [69], with the predictive median at each horizon set equal to the most recent observed weekly case count, $y_{t}$. To represent uncertainty around this median, we used the past observed changes in each state’s time series separately for each forecast horizon. Specifically, for a *h*-step-ahead forecast made at time *t*, we collected the historical *h*-step differences, $y_{s}-y_{s-h}$, and their negatives, $-(y_{s}-y_{s-h})$, for all available past times s≤t. We then formed a sampling distribution for *h*-step-ahead changes using a piecewise linear approximation to the empirical cumulative distribution function of these values. For each horizon *h*, we drew 100,000 samples from this horizon-specific distribution of *h*-step changes and added each sampled change to the last observed value $y_{t}$, yielding a Monte Carlo approximation to the *h*-step-ahead predictive distribution. The resulting simulated incidence values were truncated at zero to prevent negative forecasts. Forecast quantiles were computed from this truncated predictive distribution, with the median forced to equal $y_{t}$. For more details, see the section titled “Methods for creating baseline forecast.” in the Supplemental Materials of [69].

Empirical coverage for a (1−α)×100% forecast interval is the proportion of forecast intervals that contain the observation *y*. Empirical coverage is defined as


$$\begin{array}{c} \text{Coverage}(\alpha )=\frac{1}{N}\sum_{i=1}^{N}\text{I}(l_{i,\alpha }\leq y_{i}\leq u_{i,\alpha }) \end{array}$$ (8)

where $l_{i,\alpha }$ and $u_{i,\alpha }$ are the lower and upper quantile estimates corresponding to the (1−α)×100% forecast interval. Probabilistically well-calibrated forecasts are those whose empirical coverages are nearly equal to their nominal coverages (i.e., 1−α) for all α levels.

## Supporting information

S1 Fig MutAntiGen stochasticity. Ten MutAntiGen outputs simulated from identical input parameters.
(EPS)

S2 Fig Bootstrap comparison. Boxplots for 5,000 bootstrap samples for rMAE and rWIS from the blocked bootstrap procedure (block), taking into account state and forecast date groupings, and the vanilla bootstrap procedure (iid), which does not address groupings. Wider uncertainties are observed when taking account of the grouping variables. The white, dotted horizontal line is the point estimate for rMAE and rWIS.
(EPS)

S1 Table Initial parameter ranges of the Latin Hypercube Sample. (XLSX)

S1 Text Supplemental Material. Additional details on the MutAntiGen simulator, computational modifications, observation model, model training, and bootstrap procedures.
(PDF)
