# Supplementary material for: Leveraging synthetic and genetic data to improve epidemic forecasting
Source: PLoS Comput Biol. 2026 Aug 27;22(8):e1014630. doi: 10.1371/journal.pcbi.1014630 (PMC13521407; doi:10.1371/journal.pcbi.1014630)
Supplement: Supplementary file 1 [file pcbi.1014630.s004.pdf]

S1 Text. Supplemental Material to "Leveraging Sequence and Synthetic Data to Improve Epidemic Forecasting"

S1 Description of MutAntiGen Input Parameters

In the MutAntiGen ABM [3, 1], agents may be born, may die, may have contact with other hosts (which may or may not lead to transmission), and may recover from an infection. Each individual carries information on all viruses by which they have already been infected, as well as information about any current viral infection. The information on all current and past viral infections includes the antigenic type, the cross-immunity between antigenic types, the antigenic distance between the challenging strain and the host's history of encountered strains, probability of infection, and any immunities. Viruses may mutate in antigenic phenotype, which can create turnover and outbreak in the population when there is little cross-immunity in a population to the emergence of a new phenotype. Viruses also mutate in a "load" phenotype that affects their absolute fitness, independent of immunity in the host population.

We will now discuss in more detail the parameters that were set fixed in the MutAntiGen ABM, the parameters over which we sampled, and the justifications behind the ranges for the sampled parameters. The list of possible parameters for the MutAntiGen ABM, their descriptions, and what they were set for our simulation runs, are as follows. Parameters with an asterisk were sampled according to the sampling procedures described in the main paper and according to S1 Table (and therefore are not given a set value here).

1. burnin: Number of days to wait before logging output. This parameter was set to 0.

2. endDay: Total number of days to simulate. This parameter was set to 3650.

3. printStep: Interval in days at which output is written to `out.timeseries`. This parameter was set to 7.

4. tipSamplingStartDay: Day on which sampling for the phylogenetic tree begins, delayed to avoid basal multifurcation at time zero. This parameter was set to 56.

5. tipSamplingEndDay: Day on which sampling for the phylogenetic tree ends. This parameter was set to 3640.

6. tipSamplingRate: Sampling rate per infectious individual.

7. tipSamplesPerDeme: Maximum number of samples allowed per deme. This parameter was set to 100000.

8. tipSamplingProportional: Indicator of whether sampling is proportional to prevalence when multiple demes are present. This parameter was set to true.

9. treeProportion: Proportion of sampled tips used in tree reconstruction. This parameter was set to 1.

10. diversitySamplingCount: Number of samples drawn to compute diversity, Neτ, and serial interval. This parameter was set to 1000.

11. netauWindow: Size of the time window, in days, over which Neτ is calculated. This parameter was set to 100.

12. repeatSim: Indicator of whether the simulation is repeated until endDay is reached. This parameter was set to false.

13. immunityReconstruction: Indicator of whether immunity reconstruction output is written to `out.immunity`. This parameter was set to false.

14. memoryProfiling: Indicator of whether memory profiling is enabled, requiring a Java agent. This parameter was set to false.

15. yearsFromMK: Number of years considered as present when calculating MK statistics. This parameter was set to 1.0.

16. pcaSamples: Indicator of whether the virus tree is rotated and flipped using PCA. This parameter was set to false.

17. detailedOutput: Indicator of whether detailed host and virus output files are written to enable checkpointing. This parameter was set to false.

18. restartFromCheckpoint: Indicator of whether the population is loaded from a previous checkpoint. This parameter was set to false.

19. hostImmuneHistorySampleCount: Number of host immune histories sampled when computing mean host immunity. This parameter was set to 10000.

20. fitSampleCount: Number of viral fitness samples collected. This parameter was set to 100.

21. printFitSamplesStep: Interval in days at which viral fitness samples are printed. This parameter was set to 1000.

22. demeCount: Number of demes in the metapopulation. This parameter was set to 1.

23. demeNames: Names assigned to each deme. This parameter was set to `"tropics"`.

24. initialNs*: Initial population sizes of each deme.

25. birthRate: Per-individual daily birth rate. This parameter was set to 0.000091.

26. deathRate: Per-individual daily death rate. This parameter was set to 0.000091.

27. swapDemography: Indicator of whether overall population size is kept constant. This parameter was set to true.

28. initialDeme: Index of the deme in which infection is initiated. This parameter was set to 1.

29. initialPrR: Proportion of the population with prior immunity to the initial strain. This parameter was set to 0.5088.

30. beta*(β): Transmission rate in contacts per individual per day.

31. nu*(ν): Recovery rate in recoveries per individual per day.

32. betweenDemePro: Relative transmission rate between demes compared to within-deme transmission. This parameter was set to 0.0000.

33. externalMigration: Additional infections contributing to the force of infection. This parameter was set to 200.0.

34. transcendental: Indicator of whether a general recovered class is included. This parameter was set to false.

35. immunityLoss: Rate of immunity loss from recovered to susceptible per individual per day. This parameter was set to 0.0.

36. initialPrT: Initial fraction of the population in the general recovered class. This parameter was set to 0.0.

37. backgroundImmunity: Indicator of whether the population starts with background immunity to the current strain. This parameter was set to false.

38. backgroundDistance: Antigenic distance of background immunity from the initial strain. This parameter was set to 0.2.

39. demeBaselines: Baseline seasonal forcing for each deme. This parameter was set to `1.0`.

40. demeAmplitudes*: Amplitude of seasonal forcing for each deme.

41. demeOffsets: Seasonal phase offsets for each deme relative to the year. This parameter was set to `0.0`.

42. phenotypeSpace: Representation of phenotype space used in the model. This parameter was set to `"mutLoad"`.

43. lambda*(λ): Deleterious mutation rate per genome per transmission.

44. mutCost*: Fitness cost associated with deleterious mutations.

45. probLethal: Probability that a mutation is lethal. This parameter was set to 0.0.

46. epsilon: Beneficial mutation rate, scaled with population size. This parameter was set to 0.16.

47. epsilonSlope: Dependence of the beneficial mutation rate on mean mutational load. This parameter was set to 0.0.

48. epsilon_mut*: See discussion below.

49. initialI_prop*: See discussion below.

50. lambdaAntigenic*: Antigenic mutation rate per transmission.

51. meanAntigenicSize*: Mean size of antigenic mutations.

52. antigenicGammaShape: Shape parameter of the gamma distribution for antigenic mutation sizes. This parameter was set to 2.0.

53. thresholdAntigenicSize: Minimum antigenic mutation size required for a mutation to be retained. This parameter was set to 0.012.

54. antigenicEvoStartDay: Day on which antigenic mutations begin to occur. This parameter was set to 0.

55. cleanUpDistance: Antigenic distance beyond which strains are removed from the simulation. This parameter was set to 0.2.

56. demoNoiseScaler: Scaling factor applied to demographic noise. This parameter was set to 0.0.

57. muPhenotype: Phenotypic mutation rate per individual per day. This parameter was set to 0.0.

58. smithConversion: Multiplier converting antigenic distance into cross-immunity. This parameter was set to 0.1.

59. homologousImmunity: Immunity level conferred by antigenically identical viruses. This parameter was set to 0.95.

60. initialTraitA: Initial value of the first dimension of host immunity. This parameter was set to -6.0.

61. meanStep: Mean mutation step size. This parameter was set to 0.3.

62. sdStep: Standard deviation of mutation step size. This parameter was set to 0.3.

63. mut2D: Indicator of whether mutations occur in a full two-dimensional angular space. This parameter was set to false.

The full set of bounds for the parameters that were sampled via a Latin hs

S2 Computational Modifications to the MutAntiGen Software

There were several challenges to running a massive amount of MutAntiGen simulations over these parameter ranges. First, it is possible for a given simulation to "break" due to the current state of the system becoming untenable for the given computational resources[1]. Second, it was nearly impossible to predict how long a given simulation might take: for two simulation runs that were very similar in terms of parameter settings, one might complete fully in 30 minutes, while the other might stall out after running for 7 days. Third, not all runs showed disease turnover and behaviors of multiple antigenic mutations. Lastly, the original coding of MutAntiGen was not well-designed for running thousands of simulations in parallel on an HPC environment.

Several modifications were coded and strategies taken to extract robust and diverse synthetic data via the MutAntiGen ABM and to handle the above challenges. First, we re-coded the model to allow for multiple simulations to be run in parallel on the same HPC system; this modified version is available on this paper's GitHub page (https://github.com/lanl/precog). This re-coding also disabled some functionality of MutAntiGen that was not necessary to these experiments and was stalling out some of the simulations. Second, we created a "max wall time" that would cut simulations that did not complete within a certain time. While this cut off may bias the sampling, we did not have the computational resources to allow a given run to go for longer than 10 hours. Lastly, we performed a modified space-filling design for the selection of parameters of the ranges of interest that incorporated subject matter expertise:

1. We performed simulations over a sparse Latin-Hypercube sample (LHS) [4] of size 4000 over the parameter ranges given above. Of these 4000 samples, 815 completed.

[1] All computations were performed on an HPC cluster running Red Hat Enterprise Linux 8.10 with Slurm, using dual-socket Intel Xeon E5-2695 v3 x86_64 nodes (56 hardware threads, ∼ 256 GB RAM per node) compiled with GCC 8.5.0.

2. In order to more efficiently increase the number of simulations with significant long-term dynamics, and to ensure that outcomes with antigenic dynamics are well-represented, we identified which sets of input parameters led to simulations that represented antigenic mutation and turnover in dominant antigenic type. There were 151 sets of such input parameters, which corresponded to around 18% of the completed simulations ($\sim 3.8\%$ of the total number of simulations).

3. We performed 20 simulations at each of these input parameters, using a different seed for each input; since MutAntiGen is heavily stochastic, these replications still gave very different results. As an example, S1 Figure shows 10 of the 20 replications for a single input parameter set.

4. Of the simulations run in replicate, 1832 completed successfully. Around 65% of these completed simulations exhibited antigenic mutation and turnover in dominant antigenic type. This final set was used to train the forecasting model.

S3 Observation Model Details

Let $y_{1:T}$ be a time series of length T. In this section, we describe the observation model. That is, we describe how we stochastically convert $y_{1:T}$ into $z_{1:T'}$. For every MutAntiGen time series $y_{1:T}$, the observation model creates 20 new time series. The observation model has three steps: (1) scaling, (2) noise and (3) outliers. All 20 observation model time series undergo scaling, 10 of the observation model time series undergo noise, and each of the 20 realizations has a 25% chance to undergo outliers. Below we detail the observation model.

Scaling. We sample a new number of time steps T' for the time series $x_{1:T'}$, where

$$T' \sim \text{Uniform}(52, T)$$

(uniform over the integers). We then linearly interpolate the original time series $y_{1:T}$ to be of length T', resulting in a new time series $x_{1:T'}$. In `R` code, this is done via the `approx()` function:

```
x[1:T'] = approx(x = 1:T, y = y, xout = seq(1, T, length.out = T'))$y
```

This new time series $x_{1:T'}$ has the same dynamics as in $y_{1:T}$, but those dynamics can occur on a faster time scale than $y_{1:T}$. The smaller T', the faster the time scale. We chose this scaling approach because we were particularly concerned about underrepresenting quickly evolving outbreak dynamics, where cases can change substantially over the short forecast horizons considered here. Because we sampled $T' \leq T$, this scaling step only compressed the time axis and did not create slowed versions of the MutAntiGen outbreaks. Allowing $T' > T$ would be a natural extension of the observation model in future work.

Noise. Next, we add noise to $x_{1:T'}$, resulting in a new time series $v_{1:T'}$. The process for adding noise is as follows:

$$\kappa \sim \text{Uniform}(1.5, 3.5)$$
$$\epsilon_t|\kappa \sim \text{Uniform}(\kappa^{-1}, \kappa)$$
$$v_t = x_t * \epsilon_t$$

Note that κ is common to the time series $x_{1:T'}$ while ϵ_t is different for each element of the time series. The noise is multiplicative where $\epsilon_t \in [3.5^{-1}, 3.5]$. When κ is close to 3.5, more noise is added to the time series. When κ is close to 1.5, less noise is added. If $x_{1:T'}$ is a time series that we do not add noise to, then we simply set $v_{1:T'}$ to $x_{1:T'}$.

Outliers. Time series of public health data can have high outliers due to newly discovered data dumped onto a single date or low outliers due to non-reporting. It is important to have training data that accurately reflects real-world data, otherwise a machine learning model — having never seen outliers — may assume a large leap or drop in the time series reflects a meaningful change in the time series dynamics rather than a spurious change in the data collection process.

We randomly select between 5 and 10 time steps $\in \{1, 2, \ldots, T'\}$ to be outliers. Half of those time steps represent low outliers and the other half represent high outliers. We define multipliers λ_t for all times $t \in \{1, 2, \ldots, T'\}$ as follows:

$$\lambda_t = \begin{cases} \text{Uniform}(2, 10) & \text{if } t \text{ is a high outlier} \\ \text{Uniform}(0, 0.05) & \text{if } t \text{ is a low outlier} \\ 1 & \text{if } t \text{ is not an outlier} \end{cases}$$

Finally, we define the output time series of our observation model, $z_{1:T'}$ as follows:

$$z_t = v_t * \lambda_t.$$

10 realizations of $z_{1:T'}$ are plotted in the Materials and Methods section.

Application. To create the synthetic total cases training data, we apply the observation model to the approximately 1,800 MutAntiGen outputs where each input is recycled 20 times making 20 new $z_{1:T'}$ time series. To create the synthetic variant attributable cases training data, we sample up to ten of the variant time series and, for each one of them, we apply the scaling plus outlier routines (making one new $z_{1:T'}$ time series) and separately apply the scaling plus noise plus outlier routines (making another new $z_{1:T'}$). That is, each variant time series makes two new $z_{1:t'}$ time series. The total number of time series for training, synthetic total cases or synthetic variant attributable cases, results in about 36,000 time series.

S4 Transformer Model Details

Data and objective. We consider a collection of univariate time series, $y_{1:T}$, each provided as observations of nonnegative counts y_t ("cases") indexed by time t. Our goal is probabilistic forecasting: given the most recent $C = 20$ observations, we predict the distribution of the next $H = 4$ observations. Forecast uncertainty is represented via predictive quantiles at levels

$$\tau \in \mathcal{T} = \{0.0005, 0.005, 0.01, 0.025, 0.05, 0.1, \ldots, 0.9, 0.95, 0.975, 0.99, 0.995, 0.9995\}$$

where $\tau \in [0, 1]$ and $|\mathcal{T}| = 27$.

Training examples (rolling windows). Training uses randomly sampled rolling windows. For a series $y_{1:T}$, we repeatedly sample a start time t such that a contiguous block of length $C + H$ exists, and define the input and outputs (targets) as

$$\mathbf{y}_t^{\text{in}} = \{y_{t-C+1}, y_{t-C+2}, \ldots, y_t\}, \qquad \mathbf{y}_t^{\text{out}} = \{y_{t+1}, \ldots, y_{t+H}\}.$$

Mini-batches are formed by sampling a series with probability proportional to its number of available windows, then sampling a window uniformly within that series.

Per-window normalization and rescaling. To stabilize training across series with different magnitudes of case counts, each input window is normalized by its own maximum

$$m_t = \max(\mathbf{y}_t^{\text{in}}), \qquad \mathbf{z}_t^{\text{in}} = \mathbf{y}_t^{\text{in}}/m_t, \qquad \mathbf{z}_t^{\text{out}} = \mathbf{y}_t^{\text{out}}/m_t,$$

with the convention that if $m_t = 0$ (or is non-finite) we do not rescale that window. The model operates on $\mathbf{z}_t^{\text{in}}$ and outputs forecasts on the normalized scale. Predictions are returned to the original scale by multiplying by m_t.

Input perturbation (augmentation). To improve robustness to observation noise and related data-generating irregularities, each sampled training example is duplicated with a perturbed input context:

$$\tilde{y}_t = y_t * u_t, \qquad u_t \overset{\text{iid}}{\sim} \text{Uniform}(0.85, 1.15).$$

Thus, $\tilde{\mathbf{y}}_t^{\text{in}} = \{\tilde{y}_{t-C+1}, \tilde{y}_{t-C+2}, \ldots, \tilde{y}_t\}$ is a perturbed version of $\mathbf{y}_t^{\text{in}}$. The future targets $\mathbf{y}_t^{\text{out}}$ are unchanged. What this seeks to accomplish is a more stable learning representation. By providing the model with two distinct inputs that map to the same output, the model should be better positioned to learn stable underlying representations and less likely to learn spurious ones. This yields an effective batch size that is twice the nominal batch size.

Model architecture. We represent the forecasting function with a compact transformer encoder. Each scalar input in $\mathbf{z}_t^{\text{in}}$ is mapped to a d-dimensional embedding ($d = 256$) via a learned linear projection, and a learned positional embedding is added for the C time positions. The embedded sequence is processed by a transformer encoder with $L = 2$ layers and $n_{\text{head}} = 4$ attention heads (feedforward dimension 512). The final hidden state (at the most recent input time point) is mapped through a linear readout to produce $H \times |\mathcal{T}|$ outputs.

Quantile parameterization. The model outputs predictive quantiles $\hat{z}_{h,\tau}$ for each horizon $h = 1, \ldots, H$ and quantile level $\tau \in \mathcal{T}$. To prevent quantile crossing, quantiles are constructed using a base quantile plus nonnegative increments:

$$\hat{z}_{h,\tau_1} = a_{h,1}, \qquad \hat{z}_{h,\tau_k} = \hat{z}_{h,\tau_1} + \sum_{j=2}^{k} \text{softplus}(a_{h,j}), \quad k = 2, \ldots, |\mathcal{T}|,$$

where $a_{h,j}$ are unconstrained network outputs and $\text{softplus}(x) = \log(1 + e^x)$ ensures positivity of the increments. Forecasts on the original scale are obtained as $\hat{y}_{h,\tau} = m_t \, \hat{z}_{h,\tau}$.

Loss function (pinball loss). Parameters are estimated by minimizing the mean quantile (pinball) loss over horizons and quantile levels. For observation y_{t+h} and predicted quantile $\hat{y}_{t+h,\tau}$,

$$\rho_\tau(y_{t+h} - \hat{y}_{t+h,\tau}) = \max\big(\tau(y_{t+h} - \hat{y}_{t+h,\tau}), \, (\tau - 1)(y_{t+h} - \hat{y}_{t+h,\tau})\big),$$

and the training objective is the average of ρ_τ across all (h, τ) and all examples in each mini-batch.

Optimization, validation, and model selection. We optimize the model using Adam with a cosine-decayed learning rate schedule (from 5×10^{-4} to 5×10^{-5}) over the prescribed number of weight updates (we used 97,656). Validation uses a fixed set of randomly sampled windows (held constant by a fixed seed). We maintain an exponential moving average (EMA) of parameters with smoothing coefficient $\alpha_{\text{EMA}} = 0.98$ and select the final model as the EMA checkpoint with the lowest validation loss.

S5 Bootstrapping Details

In this study, nine different models made 27,540 forecasts (51 states/territories × 135 forecast dates × 4 forecasts horizons). Our goal is to determine which models produce forecasts that are statistically significantly better than other models. As a zeroth order pass, we can compute the metric of interest (e.g., MAE or WIS) for each model over the entire set of forecasts, and compare the numbers. Whichever model produced the best number is the best forecasting model. This, however, does not take into account uncertainty. To get an estimate of uncertainty, we turn to bootstrapping [2]. This requires us to resample the 27,540 forecasts with replacement. To deal with the groupings (e.g., states and forecast dates), we first sample states with replacement and then, for a sampled state, sample nine different blocks of time (where a block of time is 15 consecutive weeks of forecast dates). For each state/time block, we keep all horizons and all models. This results in a resampling of 27,540 forecasts per model (51 resampled states × 9 resampled time blocks × 15 dates per block × 4 horizons = 27,540 forecasts). For each resampled data set, we compute each model's MAE and WIS. We repeat this data set resampling procedure 5,000 times. The results of the block bootstrap procedure (block) and the vanilla bootstrap procedure which ignores groupings (iid) are shown in S2 Figure. Taking into account the grouping variables in the block bootstrap procedure results in wider uncertainty intervals. The 95% confidence intervals presented in the main text were derived following this block bootstrap procedure.

References

[1] Bedford, T., Rambaut, A., and Pascual, M. (2012). Canalization of the evolutionary trajectory of the human influenza virus. *BMC biology*, 10(1):38.

[2] Efron, B. and Tibshirani, R. J. (1994). *An introduction to the bootstrap*. Chapman and Hall/CRC.

[3] Koelle, K. and Rasmussen, D. A. (2015). The effects of a deleterious mutation load on patterns of influenza A/H3N2's antigenic evolution in humans. *eLife*, 4:e07361.

[4] McKay, M. D., Beckman, R. J., and Conover, W. J. (1979). A Comparison of Three Methods for Selecting Values of Input Variables in the Analysis of Output from a Computer Code. *Technometrics*, 21(2):239–245.
